# Explaining population trends in cardiovascular risk: protocol for a comparative analysis of health transitions in South Africa and England using nationally representative survey data

**DOI:** 10.1136/bmjopen-2022-061034

**Published:** 2022-03-29

**Authors:** Kafui Adjaye-Gbewonyo, Annibale Cois

**Affiliations:** 1 Faculty of Education, Health and Human Sciences, University of Greenwich, London, UK; 2 Division of Health Systems and Public Health, Stellenbosch University Faculty of Medicine and Health Sciences, Cape Town, Western Cape, South Africa; 3 Division of Epidemiology and Biostatistics, School of Public Health and Family Medicine, University of Cape Town, Cape Town, South Africa

**Keywords:** cardiac epidemiology, epidemiology, public health, social medicine

## Abstract

**Introduction:**

Cardiovascular diseases (CVD) are the leading cause of death globally and share determinants with other major non-communicable diseases. Risk factors for CVD are routinely measured in population surveys and thus provide an opportunity to study health transitions. Understanding the drivers of health transitions in countries that have not followed expected paths compared with those that exemplified models of ‘epidemiologic transition’, such as England, can generate knowledge on where resources may best be directed to reduce the burden of disease. This study aims to examine the notions of epidemiological transition by identifying and quantifying the drivers of change in CVD risk in a middle-income African setting compared with a high-income European setting.

**Methods and analysis:**

This is a secondary joint analysis of data collected within the scope of multiple population surveys conducted in South Africa and England between 1998 and 2017 on nationally representative samples of the adult population. The study will use a validated, non-laboratory risk score to estimate and compare the distribution of and trends in total CVD risk in the population. Statistical modelling techniques (fixed-effects and random-effects multilevel regression models and structural equation models) will be used to examine how various factors explain the variation in CVD risk over time in the two countries.

**Ethics and dissemination:**

This study has obtained approval from the University of Greenwich (20.5.6.8) and Stellenbosch University (X21/09/027) Research Ethics Committees. It uses anonymised microdata originating from population surveys which received ethical approval from the relevant bodies, with no additional primary data collection. Results of the study will be disseminated through (1) peer-reviewed articles in open access journals; (2) policy briefs; (3) conferences and meetings; and (4) public engagement activities designed to reach health professionals, governmental bodies, civil society and the lay public. A harmonised data set will be made publicly available through online repositories.

**Strengths and limitations of this study**

- The study assesses a composite score of cardiovascular disease risk in addition to individual risk factors.
- Trends will be examined over a nearly 20-year period and the contributions of a range of factors to these trends will be quantified at multiple levels.
- Comparative analysis will explore health transitions in different contexts.
- Source data are representative but heterogeneous.
- Innovative structural equation modelling techniques will help to account for variation across surveys.

## Introduction

### Background

In his influential paper, ‘Sick Individuals, Sick Populations’,^1^ British epidemiologist Geoffrey Rose posed two questions: ‘Why do some individuals have hypertension?’ and ‘Why do some populations have much hypertension, whilst in others it is rare?’ He wrote that while we might gain complete understanding of the first question—why individuals vary in their disease risk—we may miss ‘the most important public health question’,^1^ namely why populations have different disease burdens. As Rose noted, oftentimes what makes some individuals sicker than others is not necessarily the same as what makes some populations ‘sicker’ than others.^1^ Understanding how a population’s health is changing over time is one way to address this important second question and better understand the epidemiology of disease.

In our current era, non-communicable diseases (NCDs) have become the leading cause of death and illness globally. These conditions are often referred to as ‘chronic diseases’, having long duration, requiring continuing care and often viewed as not infectious or directly transmissible to others.^2^ In 1971, Abdul Omran proposed a theory of epidemiological transition in which societies shift from having a disease profile marked by undernutrition and infection (‘age of pestilence and famine’) to a period where these conditions decrease (‘age of receding pandemics’) and finally towards a stage dominated by chronic NCDs as life expectancies and economies improve (‘age of degenerative and man-made diseases’).^3 4^

The theory of epidemiological transition contends that this rise in NCDs such as cardiovascular disease (CVD) is caused by a number of factors. These include demographic changes, specifically the ageing of the population as mortality declines and life expectancy rises, given that the risk for many chronic diseases increases with age. In addition, decreased morbidity and mortality from infections, undernutrition and maternal and neonatal causes have also been implicated in the demographic changes leading to the rise of chronic NCDs, as medical care, sanitation and standards of living improve. ‘Lifestyle’ and behavioural changes, such as reduced physical activity and worsening diets linked to urbanisation and rising economic status, have also been hypothesised to contribute to the growing NCD ‘epidemic’. Thus, epidemiological transition is seen to parallel and be a product of several other transitions, including demographic, fertility, technological and nutritional transitions. These transitions are assumed to be occurring globally, having first occurred in industrialised, high-income countries (HICs) such as the UK while sub-Saharan African countries have typically been viewed as being at an earlier stage along this path.^3 4^

However, there have been critiques as to whether this theory is in fact a valid model of epidemiological change.^5^ Although Omran’s model has been updated over the years, many exceptions to its predictions have been noted.^4–8^ For example, a recent focus has emphasised the so-called ‘double burden of disease’ in low and middle-income countries (LMICs), representing a time in which infectious diseases are still highly prevalent, yet chronic NCDs are simultaneously rising.^6^ This is currently the case in South Africa where the HIV/AIDS and tuberculosis (TB) epidemic, combined with other communicable and nutritional diseases, NCDs and violence/injury, has even been characterised as a ‘quadruple disease burden’.^9^

The epidemiological transition theory has been criticised for being an inaccurate model of transition in many LMICs, including in the African region,^10^ and scholars such as Simon Szreter and Alexander Mercer have contended that it may not be an accurate description of the health transitions that occurred in the HICs on which the theory was built, including England.^11–13^ These and other studies suggest that ‘epidemiologic transition’ may not be the simple answer to explain changing disease patterns, underscoring the need for more detailed analyses examining other factors as well as additional research at the country level.^7^

However, empirical research to study the underlying demographic, socioeconomic and other determinants of epidemiological changes is lacking,^3^ and detailed case studies of health transition are particularly needed from the African continent.^6^

This study aims to address this need through a comparative study of South Africa and England, using CVD risk as a case study of health transitions. South Africa is a middle-income African country and a former British colony whose history of colonialism and apartheid has led to enduring structural and racial inequalities. England is an HIC whose position as a former imperial power has contributed to its comparative wealth and industrialisation. This history also ties England to South Africa. For our analysis, we draw on socioecological and ecosocial frameworks to identify potential explanatory variables of interest at the individual (biological, behavioural, socioeconomic, demographic) and contextual (environmental, societal) levels.^14 15^

This project expands on previous work by Cois examining hypertension trends in South Africa.^16–18^ In his work, he found that data from the National Income Dynamics Study (NIDS) suggested that hypertension levels have been decreasing since the late 2000s,^16^ more in line with the trends in HICs than with those in many LMICs.^19 20^ This was in spite of increasing levels of overweight/obesity and other risk factors for hypertension and was not fully explained by antihypertensive medication use.

Building on this work, this study will extend the analysis beyond blood pressure (BP) and hypertension, and examine and compare trends of overall cardiovascular risk in South Africa. The study will also attempt to identify which factors (demographic, economic, health/medical, environmental, psychosocial, behavioural) explain the changes in risk and to what extent. Thus, we will empirically test the relative contributions of factors implicated in epidemiological transition, including ageing, improved living standards, urbanisation and lifestyle change. In addition, we expand on the types of explanatory factors that have been previously examined in Cois’ work to fill in gaps and examine unanswered questions about potential drivers of change in population health. Therefore, we will incorporate demographic, behavioural, socioeconomic and healthcare variables that are traditionally considered as risk factors for CVD, and crucially we will focus on other social determinants of health that have been overlooked in the research, including psychosocial, environmental and contextual factors. We will also compare these trends in cardiovascular risk and its drivers in South Africa to those in England, where mortality from CVD has been noted to be declining in recent decades due to increased prevention and treatment.^21^

### Study aims

The aim of this study is to use existing empirical data to understand the demographic, behavioural, social and environmental drivers of recent health transitions in a middle-income African country with a high infectious disease burden compared with an HIC with a low infectious disease burden, using CVD risk as a case study. Understanding changes in population risk for chronic disease will lead to improved public health policy and prevention strategies.

The study also aims to produce a harmonised data set compiling national surveys measuring CVD risk factors in South Africa for others to use in future research as well as code for harmonising and merging the England surveys.

The research questions are as follows:

1. What are the population trends in CVD risk in South Africa since its first national health survey in 1998?
2. To what extent are these trends explained by demographic, behavioural, social, environmental, health-related and/or other factors?
3. How do these results compare to those in an HIC with a different infectious disease profile such as England over the same time period?

We hypothesise that overall CVD risk increased in South Africa and decreased in England during the study period but that these changes are not fully explained by demographic, socioeconomic, behavioural or treatment changes that often form part of epidemiological transition theory. We hypothesise that other social and environmental factors as well as infectious disease interactions may have contributed to some of the patterns in CVD risk.

## Methods and analysis

This is a secondary joint analysis of data collected within the scope of population surveys conducted in South Africa and England between 1998 and 2017 on nationally representative samples of the adult population.

### Sample and data sources

We will draw our main sample from repeated cross sections of nationally representative surveys in South Africa and England that include information on chronic disease-related conditions and risk factors, including BP readings and anthropometric measurements. Population-based surveys serve as a valuable data source to address questions regarding epidemiological change, particularly in LMICs where data from health facilities and registries are often incomplete and not representative of the wider population. National health and social surveys provide a unique opportunity to examine these questions, as they often include indicators of chronic disease risk that can easily be measured in the field such as BP and height, weight and waist circumference which can be used to measure obesity.

Data for South Africa will be drawn from the Demographic and Health Surveys (DHS), NIDS, South Africa National Health and Nutrition Examination Survey and the Study on Global Ageing and Adult Health.^22–26^ Taken together, these data sets cover 11 cross sections of the South African adult population spanning a 19-year period from 1998 to 2017. A description of the samples in these publicly available data sets is presented in table 1. Local data such as from South African Health and Demographic Surveillance Sites (HDSS) will be used to validate and inform the main analysis.^27^ In addition, we will derive area-level and environmental variables from South Africa’s censuses (1996, 2001 and 2011),^28 29^ intercensus community surveys (2007 and 2016),^29–31^ District Health Barometer^32^ and IPUMS Terra,^33^ among other sources.

**Table 1:** National surveys for analysis

South African surveys				England surveys			
Survey	Year	Adult ages	Sample size	Survey	Year	Adult ages	Sample size
DHS	1998	15+	13 827	HSE	1998	16+	15 908
				HSE	1999	16+	14 642
				HSE	2000	16+	10 481
				HSE	2001	16+	15 647
				HSE	2002	16+	10 330
DHS	2003	15+	8115	HSE	2003	16+	14 836
				HSE	2004	16+	13 520
				HSE	2005	16+	10 303
				HSE	2006	16+	14 142
SAGE	2007–2008	18+	4223	HSE	2007	16+	6882
NIDS	2008	15+	16 872	HSE	2008	16+	15 098
				HSE	2009	16+	4645
NIDS	2010–2011	15+	21 874	HSE	2010	16+	8420
NIDS	2012	15+	22 457	HSE	2011	16+	8610
SANHANES	2012	15+	7436*	HSE	2012	16+	8290
NIDS	2014–2015	15+	22 741	HSE	2013	16+	8795
SAGE	2014–2015	18+†	26 804	HSE	2014	16+	8077
				HSE	2015	16+	8034
DHS	2016	15+	5685	HSE	2016	16+	8011
NIDS	2017	15+	30 109	HSE	2017	16+	7997

*Sample completing the physical examination.

†Population representative sample aged 50+ years and a control sample aged 18–49 years.

DHS, Demographic and Health Survey; HSE, Health Survey for England; NIDS, National Income Dynamics Study; SAGE, Study on Global Ageing and Adult Health; SANHANES, South Africa National Health and Nutrition Examination Survey.

Data for England will be drawn from the Health Survey for England (HSE) over the same time period of 1998–2017 (table 1).^34 35^ Area-level variables derived from the UK Census, IPUMS Terra and other sources will be constructed to analyse the contextual effects.

All national data sets being used are publicly available from the data owners on registration/application, apart from the South African DHS 2003 which has been obtained from the National Department of Health, Special Licence data and removed variables for the HSE which are being requested from the UK Data Service and NatCen Social Research, as well as select local data sets which will be requested from the relevant HDSS.

To be consistent across surveys, we will restrict our samples to adults aged 18 years and above. However, sensitivity analyses using other age groups such as ages 16 and over or ages 50+ will also be conducted for comparison.

### Measurements

#### Outcome

The main outcome of interest (cardiovascular risk) will be quantified using Gaziano’s non-laboratory-based CVD risk score.^36^ CVD risk scores such as the Framingham Risk Score^37^ are widely used in clinical and other settings to predict the risk of having a future CVD event based on a series of laboratory results (such as lipid profiles) and other demographic and self-reported data. Non-laboratory-based measures were developed for use in low-resource settings where laboratory measures may be costly or impossible to obtain. Among those, Gaziano’s score expresses the risk of developing any CVD event in the following 5 years as a function of average systolic blood pressure (SBP), body mass index (BMI), age, sex, smoking status, current treatment for hypertension and diabetes diagnosis.^36^ It has been tested in South African samples and classified over 90% of men and over 94% of women similarly to the Framingham Risk Score.^38^ The use of alternative risk-predictive models will also be investigated prior to the final analyses. In addition, we will examine individual CVD risk components such as BP, BMI, smoking, hypertension treatment and diabetes diagnosis as secondary outcomes.

#### Explanatory variables

To assess trends in CVD risk over time, survey year will be the main time variable. To explain and decompose any time trends observed, demographic (age), socioeconomic (household assets, education, occupation), health behaviour/‘lifestyle’ (physical activity, smoking, alcohol consumption, dietary factors) and geographic (urban/rural), as well as variables relating to other health states or conditions (TB, reproductive history for women), medications and healthcare utilisation will be considered.

Given South Africa’s unique history of racial segregation and noted racial/ethnic disparities in England, racial ascription (with its historical categorisation into four socially defined ‘population groups’ in South Africa and ‘ethnicities’ in England) will also be included among the model predictors/effect modifiers as an indicator of racial discrimination. We will consider psychosocial factors by incorporating social support where available or proxies (such as marital status) and stress indicators (such as resting heart rate and household death) that are available across surveys.^39–43^

In addition, drawing on ecosocial theory and social determinants of health frameworks,^14 26^ we will incorporate environmental and contextual variables from IPUMS Terra^33^ and other local sources, such as climate data (monthly temperatures) and land use data. These will be analysed at the regional/provincial and district council levels and at smaller levels (municipality, enumeration area) where possible. We will also construct other area-level variables, such as measures of inequality, area deprivation and indicators of health services and status from censuses, community surveys, the District Health Barometer and other government sources.

### Analysis plan

Stata V.17 (StataCorp, College Station, Texas) and R statistical software V.4.0 (R Core Team, Vienna, Austria) will be used for data management, preliminary analyses and reporting. Mplus V.8 (Muthén & Muthén, Los Angeles, California) and MLwiN V.2.02 (Centre for Multilevel Modelling, Bristol, UK) will be used for path analysis, multilevel and structural modelling. Statistical analyses will be performed separately by gender, given that gender differences in CVD risk are notable^18 37^ and that reproductive factors such as pregnancy status and hormonal contraceptive use are also associated with BP and BMI. In the final stage of the study, gender-specific results will be combined to estimate to what extent changes in gender distribution across age categories and time are contributing to average population CVD risk trends.

**Figure 1:**
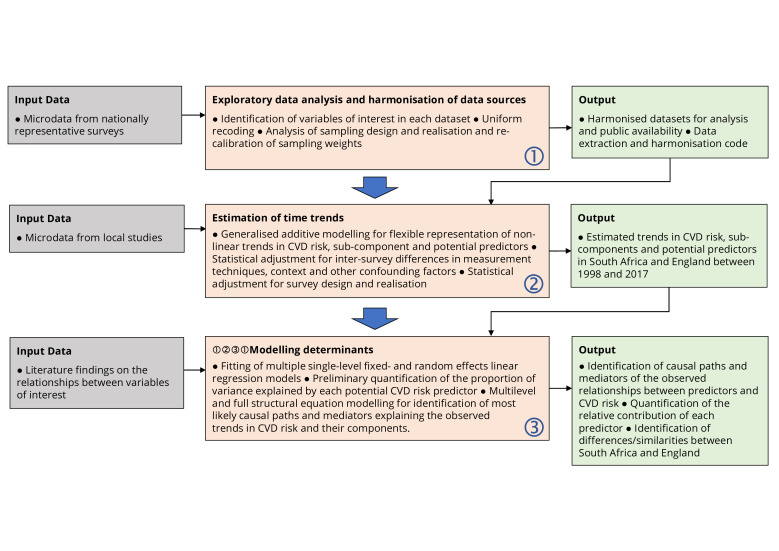
Data analysis plan. CVD, cardiovascular disease.

The pooled analysis of the available data sets will include three phases (figure 1). Our approach draws from the methods used in demography and epidemiology (public health) as well as econometric and psychometric methods:

1. In the first phase, exploratory data analysis will identify the relevant variables and ensure, with appropriate recoding and/or preliminary elaboration if necessary, the congruence of the values across data sets. The sampling designs of each survey will be analysed and compared in order to recover the information needed to produce a congruent set of pooled sampling weights and identify adequate methods to take into account the effect of clustering and stratification on the precision of the estimates. Uniform recalibration of sampling weights to match a consistent set of age-gender-specific population totals will also be considered, if justified by the observed intersurvey discrepancies.
2. In the second phase, we will recover time trends for the outcome variable (CVD risk), its subcomponents (age, SBP, smoking status, BMI, hypertension treatment, diabetes diagnosis) and the hypothesised predictors (socioeconomic, behavioural, psychosocial, infectious disease related, contextual, etc). Additive models including spline representation of the relationship of the outcome with time will be used to take into account the possibility of non-linear trends. Relevant covariates will be included to adjust for intersurvey differences in measurement and possible confounding factors (eg, seasonal differences in measurement on BP).
3. In the third phase, fixed-effects, random-effects and more general structural equation modelling (SEM) methods will be used to examine the relationships between the outcomes of interest and their hypothesised predictors in order to identify the potential determinants of the observed trends in CVD risk and its components. These methods have been chosen because they allow us to quantify and estimate the contributions of various factors to the overall variation in CVD risk over time.
  Single-level fixed-effects and random-effects linear regression models will be first used to examine the association between CVD risk, time and other potential predictors at individual level, and to quantify the proportion of variance explained by the different variables. Multilevel and full structural equation models will then be used to analyse more in detail these relationships at individual and area levels and to identify most likely causal paths and mediators explaining the observed trends in CVD risk and their components. The use of full structural models, including latent variables to represent quantities measured with error, builds and extends Dr Cois’ methodological innovations of using SEM and latent variable approaches to analyse BP while accounting for potential measurement error and variation across surveys.^17^ The approach has not been used often in health research, but is a promising method to test causal hypotheses.

In all analyses, the complex sampling design of each survey will be taken into account with the methods developed during the first phase of the study, so that (1) the estimates produced by the models will refer to the total adult populations of South Africa and England at each time point, and (2) the estimates of SEs and other measures of uncertainty are adjusted for the actual realisation of the sampling design. Missingness will be addressed through the use of both complete case analyses as well as through methods treating missingness on the outcomes as missing at random, conditional on covariates. We will run a series of models using different sets of variables. Sensitivity analyses accounting for data quality through quality scores may also be conducted.^18^

### Data and code management

A secure cloud storage system will be set up to store the original data sets as acquired from the data providers. Access to the storage area will be restricted to the study investigators to fulfil the limitations regarding data sharing as required by the data providers. Relevant metadata (including survey protocols, questionnaires and data collection procedures) will also be stored jointly with the data.

All recoding and analysis activities will be documented through Stata (.do) and/or R script files to ensure reproducibility. A GitHub (GitHub, San Francisco, California) private repository will be set up to ensure automatic versioning of all codes produced for the study and facilitate collaboration between investigators.

### Patient and public involvement

A stakeholder and user advisory group has been created for this study, consisting of members from research and academia, government, healthcare and civil society organisations. The group is providing input and guidance on the study analysis and dissemination of the findings and members of the group will be involved in the coproduction of various study outputs.

## Ethics and dissemination

### Ethics

This study has obtained ethics exemption from the University Research Ethics Committee at the University of Greenwich (reference 20.5.6.8; 20 June 2021) and approval from the Health Research Ethics Committee at Stellenbosch University (reference X21/09/027; 28 September 2021).

This project is a secondary data analysis involving the use of anonymised/deidentified microdata from population samples, and no additional primary data will be collected. All projects from which the data being used in this analysis originate received prior ethical approval from the relevant bodies and explicit consent/assent was obtained from all participants.

The study will follow the terms and conditions of the data providers, including using the data only for the purposes set out in the project, not attempting to identify individuals in the data and preserving confidentiality, citing the data sets and acknowledging and informing the data providers in and about all outputs resulting from the data. Data about the individuals in the data sets will be derived solely from the existing anonymised/deidentified data sets, and no additional information about individuals will be added to the data. Therefore, we do not foresee any added risk of identification. We will follow the framework for research ethics established by the UK Economic and Social Research Council which funds the study, including seeking to maximise the benefits and minimise the harms of the research, respecting the rights and dignity of research subjects, upholding the integrity and transparency of the research, defining lines of responsibility and accountability and maintaining the independence of the research. We will also ensure that the results of our analysis are reported accurately and responsibly.

### Dissemination

The findings of the study will be disseminated through multiple avenues, namely (1) peer-reviewed articles in open access scientific journals; (2) policy briefs for health policymakers at the national level as well as a report targeting the public sector, civil society and health professions; (3) presentations at conferences and meetings; and (4) public engagement activities and a report launch in order to reach health professionals, governmental bodies, civil society and the lay public.

Within the limitations of the data sharing agreements, a harmonised data set of the pooled national surveys for South Africa will be produced and made publicly available through major online data repositories, namely DataFirst at the University of Cape Town (https://www.datafirst.uct.ac.za/), the Human Sciences Research Council in Pretoria (http://datacuration.hsrc.ac.za/) and the UK Data Service (https://ukdataservice.ac.uk/).

The data set will include all the variables of interest used for the study, uniformly recoded to facilitate use for future studies in South Africa. To facilitate the reuse and sharing of the data, a narrative document describing all the variable definitions and derived variables created from each data set will be drafted by the investigators to accompany the data sets for deposit. This will include citations for all source data used in the creation of the data sets. The code used to harmonise and merge the HSE and South African surveys will also be made available through public repositories. A variable list and codebook will be created for both the England and South Africa’s final data sets. A series of workshops targeting researchers, students, policymakers and other subjects will be organised to provide training on the optimal use of the harmonised data sets.

## Supplementary Material

Reviewer comments

Author's manuscript
