# Supplementary material for: Explaining population trends in cardiovascular risk: protocol for a comparative analysis of health transitions in South Africa and England using nationally representative survey data
Source: BMJ Open. 2022 Mar 29;12(3):e061034. doi: 10.1136/bmjopen-2022-061034 (PMC8966565; doi:10.1136/bmjopen-2022-061034)
Supplement: Author's manuscript [file bmjopen-2022-061034.draft_revisions.pdf]

BMJ Open

BMJ Open is committed to open peer review. As part of this commitment we make the peer review history of every article we publish publicly available.

When an article is published we post the peer reviewers' comments and the authors' responses online. We also post the versions of the paper that were used during peer review. These are the versions that the peer review comments apply to.

The versions of the paper that follow are the versions that were submitted during the peer review process. They are not the versions of record or the final published versions. They should not be cited or distributed as the published version of this manuscript.

BMJ Open is an open access journal and the full, final, typeset and author-corrected version of record of the manuscript is available on our site with no access controls, subscription charges or pay-per-view fees (http://bmjopen.bmj.com).

If you have any questions on BMJ Open's open peer review process please email info.bmjopen@bmj.com

BMJ Open

Explaining population trends in cardiovascular risk: A comparative analysis of health transitions in South Africa and England

Journal:	*BMJ Open*
Manuscript ID	bmjopen-2022-061034
Article Type:	Protocol
Date Submitted by the Author:	13-Jan-2022
Complete List of Authors:	Adjaye-Gbewonyo, Kafui; University of Greenwich,<br>Cois, Annibale; Stellenbosch University Faculty of Medicine and Health Sciences, Division of Helath systems and Public Health
Keywords:	Cardiac Epidemiology < CARDIOLOGY, EPIDEMIOLOGY, PUBLIC HEALTH, SOCIAL MEDICINE

SCHOLARONE™
Manuscripts

Title

Explaining population trends in cardiovascular risk: A comparative analysis of health transitions in South Africa and England

Kafui Adjaye-Gbewonyo[1], Annibale Cois[2]

[1] Institute for Lifecourse Development, Faculty of Education, Health and Human Sciences, School of Human Sciences, University of Greenwich, London, UK.

[2] Division of Health Systems and Public Health, Department of Global Health, Stellenbosch University, Tygerberg, South Africa

Corresponding Author: Dr Kafui Adjaye-Gbewonyo, K.AdjayeGbewonyo@greenwich.ac.uk, Faculty of Education, Health and Human Sciences, School of Human Sciences, Dreadnought Building, Old Royal Naval College, Park Row, London, SE10 9LS

Word count: 3733

For peer review only

Abstract

Introduction Cardiovascular diseases (CVD) are the leading causes of death globally and share determinants with other major non-communicable diseases. Risk factors for CVD are routinely measured in population surveys and thus provide an opportunity to study health transitions. Understanding the drivers of health transitions in countries that have not followed expected paths compared to those that exemplified models of 'epidemiologic transition', such as England, can generate knowledge on where resources may best be directed to reduce the burden of disease.

This study aims to examine notions of epidemiologic transition by identifying and quantifying the drivers of change in CVD risk in a middle-income African setting compared to a high-income European setting.

Methods and analysis This is a secondary joint analysis of data collected within the scope of multiple population surveys conducted in South Africa and England between 1998 and 2017 on nationally-representative samples of the adult population. The study will use a validated non-laboratory risk score to estimate and compare the distribution of and trends in total CVD risk in the population. Statistical modelling techniques (fixed-effects and random-effects multilevel regression models and structural equation models) will be used to examine how various factors explain the variation in CVD risk over time in the two countries.

Ethics and dissemination This study has obtained approval from the University of Greenwich (20.5.6.8) and Stellenbosch University (X21/09/027) Research Ethics Committees. It uses anonymised microdata originating from population surveys which received ethical approval by the relevant bodies, with no additional primary data collection.

Results of the study will be disseminated through (1) peer-reviewed articles in open access journals; (2) policy briefs; (3) conferences and meetings; (4) and public engagement activities in order to reach health professionals, governmental bodies, civil society and the lay public. A harmonised dataset will be made publicly available through online repositories.

Strengths and limitations of this study

- The study assesses a composite score of CVD risk in addition to individual risk factors.
- Trends will be examined over a nearly 20-year period and the contributions of a range of factors to these trends will be quantified at multiple levels.
- Comparative analysis will explore health transitions in different contexts.
- Innovative structural equation modelling techniques help to account for variation across surveys.
- Source data are representative but heterogeneous.

For peer review only

INTRODUCTION

Background

In his influential paper 'Sick Individuals, Sick Populations',[1] British epidemiologist Geoffrey Rose posed two questions: "*Why do some individuals have hypertension?*" and "*Why do some populations have much hypertension, whilst in others it is rare?*" He wrote that while we might gain complete understanding of the first question—why individuals vary in their disease risk—we may miss "*the most important public health question*", namely why populations have different disease burdens. As Rose noted, oftentimes what makes some individuals sicker than others is not necessarily the same as what makes some populations 'sicker' than others.[1] Understanding how a population's health is changing over time is one way to address this important second question and better understand the epidemiology of disease.

In our current era, non-communicable diseases (NCDs) have become the leading causes of death and illness globally. These conditions are often referred to as 'chronic diseases', having long duration, requiring continuing care, and often viewed as not infectious or directly transmissible to others.[2] In 1971, Abdul Omran proposed a theory of epidemiologic transition, in which societies shift from having a disease profile marked by undernutrition and infection ("*age of pestilence and famine*") to a period where these conditions decrease ("*age of receding pandemics*") and finally toward a stage dominated by chronic NCDs as life expectancies and economies improve ("*age of degenerative and man-made diseases*").[3,4]

The theory of epidemiologic transition contends that this rise in NCDs such as cardiovascular disease (CVD) is caused by a number of factors. These include demographic changes, specifically the ageing of the population as mortality declines and life expectancy rises, given that the risk for many chronic diseases increases with age. In addition, decreased morbidity and mortality from infections, undernutrition and maternal and neonatal causes have also been implicated in the demographic changes leading to the rise of chronic NCDs as medical care, sanitation and standards of living improve. 'Lifestyle' and behavioural changes such as reduced physical activity and worsening diets linked to urbanisation and rising economic status, have also been hypothesised to contribute to the growing NCD 'epidemic'. Thus, epidemiologic transition is seen to parallel and be a product of several other transitions, including demographic, fertility, technologic and nutritional transitions. These transitions are assumed to be occurring globally, having first occurred in industrialised, high-income countries (HICs) such as the UK while Sub-Saharan African countries have typically been viewed as being at an earlier stage along this path.[3,4]

However, there have been critiques as to whether this theory is in fact a valid model of epidemiologic change.[5] Although the Omran's model has been updated over the years, many exceptions to its predictions have been noted.[4-7] For example, recent focus has emphasized the so-called 'double burden of disease' in low- and middle-income countries (LMICs), representing a time in which infectious diseases are still highly prevalent, yet chronic NCDs are simultaneously rising.[6] This is currently the case in South Africa where the HIV/AIDS and tuberculosis (TB) epidemic, combined with other communicable and nutritional diseases, NCDs and violence/injury has even been characterised as a 'quadruple disease burden'.[9]

Not only has the epidemiologic transition theory been criticised for being an inaccurate model of transition in many LMICs, including in the African region,[10] scholars such as Simon Szreter and Alexander Mercer have also contended that it may not be an accurate description of the health transitions that occurred in the HICs on which the theory was built, including England.[11-13] These

For peer review only

and other studies suggest that 'epidemiologic transition' may not be the simple answer to explain changing disease patterns, underscoring the need for more detailed analyses examining other factors as well as additional research at the country level.[7]

However, empirical research to study the underlying demographic, socio-economic and other determinants of epidemiological changes is lacking,[3] and detailed case studies of health transition are particularly needed from the African continent.[6]

This study aims to address this need through a comparative study of South Africa and England, using CVD risk as a case study of health transitions. South Africa is a middle-income African country and former British colony whose history of colonialism and apartheid has led to enduring structural and racial inequalities. England is a high-income country whose position as a former imperial power has contributed to its comparative wealth and industrialisation. This history also ties England to South Africa. For our analysis, we draw on socio-ecological and ecosocial frameworks to identify potential explanatory variables of interest at the individual (biological, behavioural, socio-economic, demographic) and contextual (environmental, societal) levels.[14,15]

This project expands on previous work by Cois examining hypertension trends in South Africa. [16-18] In his work, he found that data from the National Income Dynamics Study suggested that hypertension levels have been decreasing since the late 2000s,[16] more in line with the trends in HICs than with those in many LMICs.[19,20] This was in spite of increasing levels of overweight/obesity and other risk factors for hypertension and was not fully explained by antihypertensive medication use.

Building on this work, this study will extend the analysis beyond blood pressure (BP) and hypertension, and examine and compare trends of overall cardiovascular risk in South Africa. The study will also attempt to identify which factors (demographic, economic, health/medical, environmental, psychosocial, behavioural) explain the changes in risk and to what extent. Thus, we will empirically test the relative contributions of factors implicated in epidemiologic transition, including ageing, improved living standards, urbanisation, lifestyle change. In addition, we expand on the types of explanatory factors that have been previously examined in Cois's work to fill in gaps and examine unanswered questions about potential drivers of change in population health. Therefore, we will incorporate not only demographic, behavioural, socioeconomic and healthcare variables that are traditionally considered as risk factors for CVD, but crucially we will also focus on other social determinants of health that have been overlooked in the research, including psychosocial, environmental and contextual factors. We will also compare these trends in cardiovascular risk and its drivers in South Africa to those in England, where mortality from CVD has been noted to be declining in recent decades due to increased prevention and treatment [21].

Study Aims

The aim of this study is to utilise existing empirical data to understand the demographic, behavioural, social and environmental drivers of recent health transitions in a middle-income African country with a high infectious disease burden compared to a HIC with a low infectious disease burden, using CVD risk as a case study. Understanding changes in population risk for chronic disease will lead to improved public health policy and prevention strategies.

The study also aims to produce a harmonised dataset compiling national surveys measuring CVD risk factors in South Africa for others to use in future research as well as code for harmonising and merging the England surveys.

The research questions are as follows:

For peer review only - http://bmjopen.bmj.com/site/about/guidelines.xhtml

1. What are the population trends in CVD risk in South Africa since its first national health survey in 1998?
2. To what extent are these trends explained by demographic, behavioural, social, environmental, health-related and/or other factors?
3. How do these results compare to those in a high-income country with a different infectious disease profile such as England over the same time period?

We hypothesise that overall CVD risk increased in South Africa during the study period but that this increase is not fully explained by demographic, socioeconomic, behavioural or treatment changes that often form part of epidemiological transition theory. We hypothesise that other social and environmental factors as well as infectious disease interactions may have contributed to some of the patterns in CVD risk.

METHODS AND ANALYSIS

This is a secondary joint analysis of data collected within the scope of populations surveys conducted in South Africa and England between 1998 and 2017 on nationally representative samples of the adult population.

Sample and data sources

We will draw our main sample from repeated cross-sections of nationally-representative surveys in South Africa and England that include information on chronic-disease related conditions and risk factors, including BP readings and anthropometric measurements. Population-based surveys serve as a valuable data source to address questions regarding epidemiologic change, particularly in LMICs where data from health facilities and registries is often incomplete and not representative of the wider population. National health and social surveys provide a unique opportunity to examine these questions, as they often include indicators of chronic disease risk that can easily be measured in the field such as BP and height, weight and waist circumference which can be used to measure obesity.

Data for South Africa will be drawn from the Demographic and Health Surveys (DHS), National Income Dynamics Study (NIDS), South Africa National Health and Nutrition Examination Survey (SANHANES) and Study on Global Ageing and Adult Health (SAGE).[22-26] Taken together, these datasets cover 11 cross-sections of the South African adult population spanning a 19-year period from 1998 to 2017. A description of the samples in these publicly-available datasets is presented in Table 1. Local data such as from South African Health and Demographic Surveillance Sites (HDSSs) will be used to validate and inform the main analysis.[27] In addition, we will derive area-level and environmental variables from South Africa's censuses (1996, 2001 and 2011),[28,29] inter-census Community Surveys (2007 and 2016),[29-31], District Health Barometer [32] and IPUMS Terra[33], among other sources.

Data for England will be drawn from the Health Survey for England (HSE) over the same time period of 1998 to 2017 (Table 1).[34,35] Area-level variables derived from the UK Census, IPUMS Terra and other sources will be constructed to analyse contextual effects.

For peer review only - http://bmjopen.bmj.com/site/about/guidelines.xhtml

Table 1. National Surveys for Analysis

South African Surveys				England Surveys			
**Survey**	**Year**	**Adult Ages**	**Sample Size**	**Survey**	**Year**	**Adult Ages**	**Sample Size**
DHS	1998	15+	13 827	HSE	1998	16+	15 908
				HSE	1999	16+	14 642
				HSE	2000	16+	10 481
				HSE	2001	16+	15 647
				HSE	2002	16+	10 330
DHS	2003	15+	8 115	HSE	2003	16+	14 836
				HSE	2004	16+	13 520
				HSE	2005	16+	10 303
				HSE	2006	16+	14 142
SAGE	2007-8	18+	4 223	HSE	2007	16+	6 882
NIDS	2008	15+	16 872	HSE	2008	16+	15 098
				HSE	2009	16+	4 645
NIDS	2010-11	15+	21 874	HSE	2010	16+	8 420
NIDS	2012	15+	22 457	HSE	2011	16+	8 610
SANHANES	2012	15+	7 436 [a]	HSE	2012	16+	8 290
NIDS	2014-15	15+	22 741	HSE	2013	16+	8 795
SAGE	2014-15	18+ [b]	26 804	HSE	2014	16+	8 077
				HSE	2015	16+	8 034
DHS	2016	15+	5 685	HSE	2016	16+	8 011
NIDS	2017	15+	30 109	HSE	2017	16+	7 997

[a] Sample completing the physical exam; [b] Population-representative sample 50+, and a control sample 18-49 years.

HSE = Health Survey for England; DHS = Demographic and Health Survey; SAGE = Study on Global Ageing and Adult Health; NIDS = National Income Dynamics Study; SANHANES = South Africa National Health and Nutrition Examination Survey.

All national datasets being used are publicly available from the data owners upon registration/ application, apart from the South African DHS 2003 which has been obtained from the National Department of Health, Special Licence data and removed variables for the HSE which are being requested from the UK Data Service and NatCen Social Research, as well as select local datasets which will be requested from the relevant HDSSs.

To be consistent across surveys, we will restrict our samples to adults aged 18 years and above. However, sensitivity analyses using other age groups such as ages 16 and over or ages 50+ will also be conducted for comparison.

Measurements

Outcome

The main outcome of interest (cardiovascular risk) will be quantified using Gaziano's non-laboratory based CVD risk score.[36] CVD risk scores such as the Framingham risk scores [37] are widely used in clinical and other settings to predict risk of having a future CVD event based on a series of laboratory results (such as lipid profiles) and other demographic and self-reported data. Non-laboratory-based measures were developed for use in low-resource settings where laboratory measures may be costly or impossible to obtain. Among those, Gaziano's score expresses the risk of developing any

For peer review only

cardiovascular disease event in the following 5-years as a function of average systolic blood pressure (SBP), body mass index (BMI), age, sex, smoking status, current treatment for hypertension and diabetes diagnosis.[36] It has been tested in South African samples and classified over 90% of men and over 94% of women similarly to the Framingham risk score.[38] The use of alternative risk predictive models will also be investigated prior to the final analyses. In addition, we will examine individual CVD risk components such as BP, BMI, smoking hypertension treatment and diabetes diagnosis as secondary outcomes.

Explanatory variables

To assess trends in CVD risk over time, survey year will be the main time variable. To explain and decompose any time trends observed, demographic (age), socioeconomic (household assets, education, occupation, household size), health behaviour/'lifestyle' (physical activity, smoking, alcohol consumption, dietary factors) and geographic (urban/rural), as well as variables relating to other health states or conditions (TB, reproductive history for women), medications and healthcare utilisation will be considered.

Given South Africa's unique history of racial segregation and noted racial/ethnic disparities in England, racial ascription (with its historical categorisation into four socially defined 'population groups' in South Africa and 'ethnicities' in England) will be also be included among the model predictors/effect modifiers as an indicator of racial discrimination. We will consider psychosocial factors by incorporating social support where available or proxies (such as marital status) and stress indicators (such as resting heart rate and household death) that are available across surveys.[39-43]

In addition, drawing on ecosocial theory and social determinants of health frameworks,[14,26] we will incorporate environmental and contextual variables from IPUMS Terra[33] and other local sources, such as climate data (monthly temperatures) and land use data. These will be analysed at the regional/provincial and district council levels and at smaller levels (municipality, enumeration area) where possible. We will also construct other area-level variables, such as measures of inequality, area deprivation and indicators of health services and status from censuses, community surveys, the District Health Barometer and other government sources.

Analysis plan

Stata v. 17 (StataCorp, College Station, TX) and R Statistical software v. 4.0 (R Core Team, Vienna, Austria) will be used for data management, preliminary analyses and reporting. Mplus v. 8 (Muthén & Muthén, Los Angeles, CA) and MLwiN v. 2.02 (Centre for Multilevel Modelling, Bristol, UK) will be used for path analysis, multilevel and structural modelling. Statistical analyses will be performed separately by gender, given that gender differences in CVD risk are notable[18, 37] and that reproductive factors such as pregnancy status and hormonal contraceptive use are also associated with BP and BMI. In the final stage of the study, gender-specific results will be combined to estimate to what extent changes in gender distribution across age categories and time are contributing to average population CVD risk trends.

The pooled analysis of the available datasets will include three phases (Figure 1). Our approach draws from methods used in demography and epidemiology (public health) as well as econometric and psychometric methods:

1. In the first phase, exploratory data analysis will identify the relevant variables and ensure, with appropriate recoding and/or preliminary elaboration if necessary, the congruence of the values across datasets. The sampling designs of each individual survey will be analysed and compared, in order to recover the information needed to produce a congruent set of pooled sampling weights

For peer review only

and identify adequate methods to take into account the effect of clustering and stratification on the precision of the estimates. Uniform re-calibration of sampling weights in order to match a consistent set of age-gender-specific population totals will be also considered, if justified by the observed inter-survey discrepancies.

2. In the second phase, we will recover time trends for the outcome variable (CVD risk), its sub-components (age, SBP, smoking status, BMI, hypertension treatment, diabetes diagnosis) and the hypothesised predictors (socioeconomic, behavioural, psychosocial, infectious-disease-related, contextual, etc.). Additive models including spline representation of the relationship of the outcome with time will be used to take into account the possibility of non-linear trends. Relevant covariates will be included to adjust for inter-survey differences in measurement and possible confounding factors (e.g. seasonal differences in measurement on blood pressure)

3. In the third phase, fixed-effects, random-effects and more general structural equation modelling (SEM) methods will be used to examine the relationships between the outcomes of interest and their hypothesised predictors, in order to identify potential determinants of the observed trends in CVD risk and its components. These methods have been chosen because they allow us to quantify and estimate the contributions of various factors to the overall variation in CVD risk over time.

   Single-level fixed- and random effects linear regression models will be first used to examine the association between CVD risk, time and other potential predictors at individual level, and to quantify the proportion of variance explained by the different variables. Multilevel and full structural equation models will be then used to analyse more in details these relationships at individual and area-levels and to identify most likely causal paths and mediators explaining the observed trends in CVD risk and their components. The use of full structural models, including latent variables to represent quantities measured with error builds and extends Dr Cois's methodological innovations of using SEM and latent variable approaches to analyse BP while accounting for potential measurement error and variation across surveys.[17] The approach has not been used often in health research, but is a promising method to test causal hypotheses.

In all analyses, the complex sampling design of each survey will be taken into account with the methods developed during the first phase of the study, so that 1) the estimates produced by the models will refer to the total populations of South Africa and England at each time point, and 2) the estimates of standard errors and other measures of uncertainty are adjusted for the actual realisation of the sampling design. Missingness will be addressed through the use of both complete-case analyses as well as through methods treating missingness on the outcomes as missing at random conditional on covariates. We will run a series of models using different sets of variables. Sensitivity analyses accounting for data quality through quality scores may also be conducted.[18]

Data and code management

A secure cloud storage system will be set up in order to store the original datasets as acquired from the data providers. Access to the storage area will be restricted to the study investigators, in order to fulfil limitations regarding data sharing as required by the data provider. Relevant metadata (including survey protocols, questionnaires and data collection procedures) will also be stored jointly with the data.

All recoding and analysis activities will be documented though Stata (.do) and/or R script files, to ensure reproducibility. A GitHub (GitHub Inc, San Francisco, CA) private repository will be set up to ensure automatic versioning of all code produced for the study and facilitate collaboration between investigators.

For peer review only

Patient and public involvement

A stakeholder and user advisory group has been created for this study, consisting of members from research and academia, government, healthcare and civil society organisations. The group is providing input and guidance on the study analysis and dissemination of the findings and members of the group will be involved in the co-production of various study outputs.

ETHICS AND DISSEMINATION

Ethics

This study has obtained Ethics exemption from the University Research Ethics Committee at the University of Greenwich (Ref. 20.5.6.8, 20/06/2021) and approval from the Health Research Ethics Committee at Stellenbosch University (Ref. X21/09/027, 28/09/2021).

This project is a secondary data analysis involving the use of anonymised/de-identified microdata from population samples, and no additional primary data will be collected. All projects from which the data being used in this analysis originate received prior ethical approval by the relevant bodies and explicit consent/assent was obtained from all participants.

The study will follow the terms and conditions of the data providers, including using the data only for the purposes set out in the project, not attempting to identify individuals in the data and preserving confidentiality, citing the datasets and acknowledging and informing the data providers in and about all outputs resulting from the data. Data about the individuals in the datasets will be derived solely from the existing anonymised/de-identified datasets, and no additional information about individuals will be added to the data. Therefore, we do not foresee any added risk of identification. We will follow the framework for research ethics established by the UK Economic and Social Research Council (ESRC) which funds the study, including seeking to maximise the benefits and minimise the harms of the research, respecting the rights and dignity of research subjects, upholding the integrity and transparency of the research, defining lines of responsibility and accountability and maintaining the independence of the research. We will also ensure that results of our analysis are reported accurately and responsibly.

Dissemination

The findings of the study will be disseminated through multiple avenues, namely (1) peer-reviewed articles in open access scientific journals; (2) policy briefs for health policymakers at the national level as well as a report targeting the public sector, civil society and health professions; (3) presentation at conferences and meetings; (4) public engagement activities and a report launch in order to reach health professionals, governmental bodies, civil society and the lay public.

Within the limitations of the data sharing agreements, a harmonised dataset of the pooled national surveys for South Africa which will be produced and made publicly available through major online data repositories, namely DataFirst at the University of Cape Town (https://www.datafirst.uct.ac.za/), the Human Sciences Research Council in Pretoria (http://datacuration.hsrc.ac.za/) and the UK Data service (https://ukdataservice.ac.uk/).

The dataset will include all the variables of interest used for the study, uniformly recoded to facilitate use for future studies in South Africa. To facilitate the re-use and sharing of the data, a narrative document describing all the variable definitions and derived variables created from each dataset will be drafted by the investigators to accompany the datasets for deposit. This will include citations for

For peer review only

all source data used in the creation of the datasets. The code used to harmonise and merge the Health Surveys for England and South African surveys will also be made available through public repositories. A variable list and codebook will be created for both the England and South Africa final datasets. A series of workshops targeting researchers, students, policymakers and other subjects will be organised to provide training on the optimal use of the harmonised datasets.

REFERENCES

1. Rose G. Sick individuals and sick populations. *Int J Epidemiol* 2001;30(3):427-32.
2. Adjaye-Gbewonyo K, Vaughan M. Reframing NCDs? An analysis of current debates. *Glob Health Action* 2019;12(1), DOI: 10.1080/16549716.2019.1641043.
3. Santosa A, Wall S, Fottrell E, et al. The development and experience of epidemiological transition theory over four decades: a systematic review. *Glob Health Action* 2014;7:23574.
4. Omran AR. The Epidemiologic Transition: A Theory of the Epidemiology of Population Change. *Milbank Mem Fund Q* 1971;49(4):509-38.
5. Vaughan, M, Adjaye-Gbewonyo, K, Mika, M (eds.). Epidemiological Change and Chronic Disease in Sub-Saharan Africa: Social and historical perspectives. London: UCL Press; 2021 [Available from: https://doi.org/10.14324/111.9781787357044]
6. Agyei-Mensah S, de-Graft Aikins A. Epidemiological transition and the double burden of disease in Accra, Ghana. *J Urban Health* 2010;87(5):879-97.
7. Murray CJL, Barber RM, Foreman KJ, et al. Global, regional, and national disability-adjusted life years (DALYs) for 306 diseases and injuries and healthy life expectancy (HALE) for 188 countries, 1990–2013: quantifying the epidemiological transition. *The Lancet* 2015;386(10009):2145-91.
8. Kabudula CW, Houle B, Collinson MA, et al. Progression of the epidemiological transition in a rural South African setting: findings from population surveillance in Agincourt, 1993-2013. *BMC Public Health* 2017;17(1):424.
9. Pillay-van Wyk V, Msemburi W, Laubscher R, et al. Mortality trends and differentials in South Africa from 1997 to 2012: second National Burden of Disease Study. *The Lancet Global Health* 2016;4(9):e642-e53.
10. Vaughan M. Conceptualising metabolic disorder in Southern Africa: Biology, history and global health. *BioSocieties* 2018;14(1):123–42.
11. Szreter S, Mooney G. Urbanization, Mortality, and the Standard of Living Debate: New Estimates of the Expectation of Life at Birth in Nineteenth-century British Cities. *The Economic History Review* 1998;51(1):84-112.
12. Szreter S. Industrialization and health. *Br Med Bull* 2004;69:75-86.
13. Mercer A. Infections, chronic disease, and the epidemiological transition: A new perspective. Rochester: University of Rochester Press; 2014.
14. Krieger N. Proximal, distal, and the politics of causation: What's level got to do with it? *American Journal of Public Health* 2008;98(2):221-30.

For peer review only

15. Krieger N. Epidemiology and the people's health: Theory and context. New York: Oxford University Press; 2011.

16. Cois A, Ehrlich R. Antihypertensive treatment and blood pressure trends among South African adults: A repeated cross-sectional analysis of a population panel survey. *PLoS One* 2018;13(8):e0200606.

17. Cois A, Ehrlich R. Analysing the socioeconomic determinants of hypertension in South Africa: a structural equation modelling approach. *BMC Public Health* 2014;14:414.

18. Cois A. Understanding blood pressure dynamics in the South African population: a latent variables approach to the analysis and comparison of data from multiple surveys (Doctoral dissertation]. Cape Town: University of Cape Town; 2017. [Available from: https://open.uct.ac.za/handle/11427/25196]

19. Danaei G, Finucane MM, Lin JK, et al. National, regional, and global trends in systolic blood pressure since 1980: systematic analysis of health examination surveys and epidemiological studies with 786 country-years and 5·4 million participants. *The Lancet* 2011;377(9765):568-77.

20. Zhou B, Bentham J, Di Cesare M, et al. Worldwide trends in blood pressure from 1975 to 2015: a pooled analysis of 1479 population-based measurement studies with 19.1 million participants. *The Lancet* 2017;389(10064):37-55.

21. Guzman-Castillo M, Ahmed R, Hawkins N, et al. The contribution of primary prevention medication and dietary change in coronary mortality reduction in England between 2000 and 2007: a modelling study. *BMJ Open* 2015;5:e006070.

22. Department of Health Republic of South Africa. South Africa Demographic and Health Survey 1998: Full Report. Pretoria: Department of Health; 2002.

23. National Income Dynamics Study. Website. [Available from: http://www.nids.uct.ac.za. Accessed: October 2021].

24. Shisana O, Labadarios D, Rehle T, et al. South African National Health and Nutrition Examination Survey (SANHANES-1). Cape Town: HSRC Press; 2013.

25. World Health Organization. Health statistics and information systems: WHO Study on global AGEing and adult health (SAGE). [Available from: http://www.who.int/healthinfo/sage/en/. Accessed: October 2021]

26. Kowal P, Chatterji S, Naidoo N, et al. Data resource profile: the World Health Organization Study on global AGEing and adult health (SAGE). *Int J Epidemiol* 2012;41(6):1639-49.

27. SAPRIN. South African Population Research Infrastructure Network [Available from: http://saprin.mrc.ac.za/about.html. Accessed: October 2021]

28. Statistics South Africa. Census 2011 Statistical Release - P0301.4. Pretoria: Stats SA; 2012.

29. Minnesota Population Center. Integrated Public Use Microdata Series, International: Version 7.2 [Machine-Readable Database]. Minneapolis: University of Minnesota; 2019.

30. Statistics South Africa. Community Survey, 2007 (revised version). Pretoria: Stats SA; 2007.

31. Statistics South Africa. Community Survey 2016, Statistical release P0301. Pretoria: Stats SA; 2016.

For peer review only

32. Massyn N, Tanna G, Day C, et al. District Health Barometer: District Health Profiles 2017/18. Durban: Health Systems Trust; 2018.

33. Ruggles S, Manson SM, Kugler TA, et al. IPUMS Terra: Integrated Data on Population and Environment. 2 ed. Minneapolis, MN: IPUMS; 2018.

34. Health Survey for England London: UCL; 2019 [Available from: https://www.ucl.ac.uk/epidemiology-health-care/research/epidemiology-and-public-health/research/health-and-social-surveys-research-group/studies-0. Accessed: October 2021]

35. Mindell J, Biddulph JP, Hirani V, et al. Cohort profile: the health survey for England. *Int J Epidemiol* 2012;41(6):1585-93.

36. Gaziano TA, Young CR, Fitzmaurice G, et al. Laboratory-based versus non-laboratory-based method for assessment of cardiovascular disease risk: the NHANES I Follow-up Study cohort. *The Lancet* 2008;371(9616):923-31.

37. Damen JA, Pajouheshnia R, Heus P, et al. Performance of the Framingham risk models and pooled cohort equations for predicting 10-year risk of cardiovascular disease: a systematic review and meta-analysis. *BMC Med* 2019;17(109).

38. Gaziano TA, Pandya A, Steyn K, et al. Comparative assessment of absolute cardiovascular disease risk characterization from non-laboratory-based risk assessment in South African populations. *BMC Med* 2013;11:170.

39. Adjaye-Gbewonyo K, Avendano M, Subramanian SV, et al. Income inequality and depressive symptoms in South Africa: A longitudinal analysis of the National Income Dynamics Study. *Health & Place* 2016;42:37-46.

40. Adjaye-Gbewonyo K, Kawachi I, Subramanian SV, et al. High social trust associated with increased depressive symptoms in a longitudinal South African sample. *Social Science & Medicine* 2018;197:127-35.

41. Adjaye-Gbewonyo K, Kawachi I, Subramanian SV, et al. Income inequality and cardiovascular disease risk factors in a highly unequal country: a fixed-effects analysis from South Africa. *Int J Equity Health* 2018;17(1):31.

42. Cois A, Day C. Obesity trends and risk factors in the South African adult population. *BMC Obesity* 2015;2:42.

43. Cois A, Ehrlich R. Socioeconomic Status Modifies the Seasonal Effect on Blood Pressure: Findings from a National Panel Study. *Medicine* 2015;94(35):e1389.

Acknowledgments

The authors thank Megan Vaughan (UCL), Ed Fottrell (UCL), Luis Rego (UCL), Debbie Bradshaw (SAMRC) and Jané Joubert (SAMRC) for their valuable comments on drafts of this protocol.

Authors’ contributions

The study was conceived by KA, who prepared the first draft of the protocol. AC contributed in refining the scope of the study, and the analysis, dissemination and data sharing plans. Both authors read and

For peer review only

approved the final protocol. KA and AC will conduct variable selection and harmonisation across datasets; implement the analysis strategy and run the analyses; draft and revise reports/publications resulting from the analyses; implement research impact and stakeholder involvement activities. KA will ensure overall coordination of the research activities as the PI of the study, in collaboration with AC as the co-PI.

Funding

This work is supported by UKRI Economic and Social Research Council grant number ES/V003259/1.

Competing interests

None declared.

For peer review only

FIGURE CAPTIONS

Figure 1: Data analysis plan

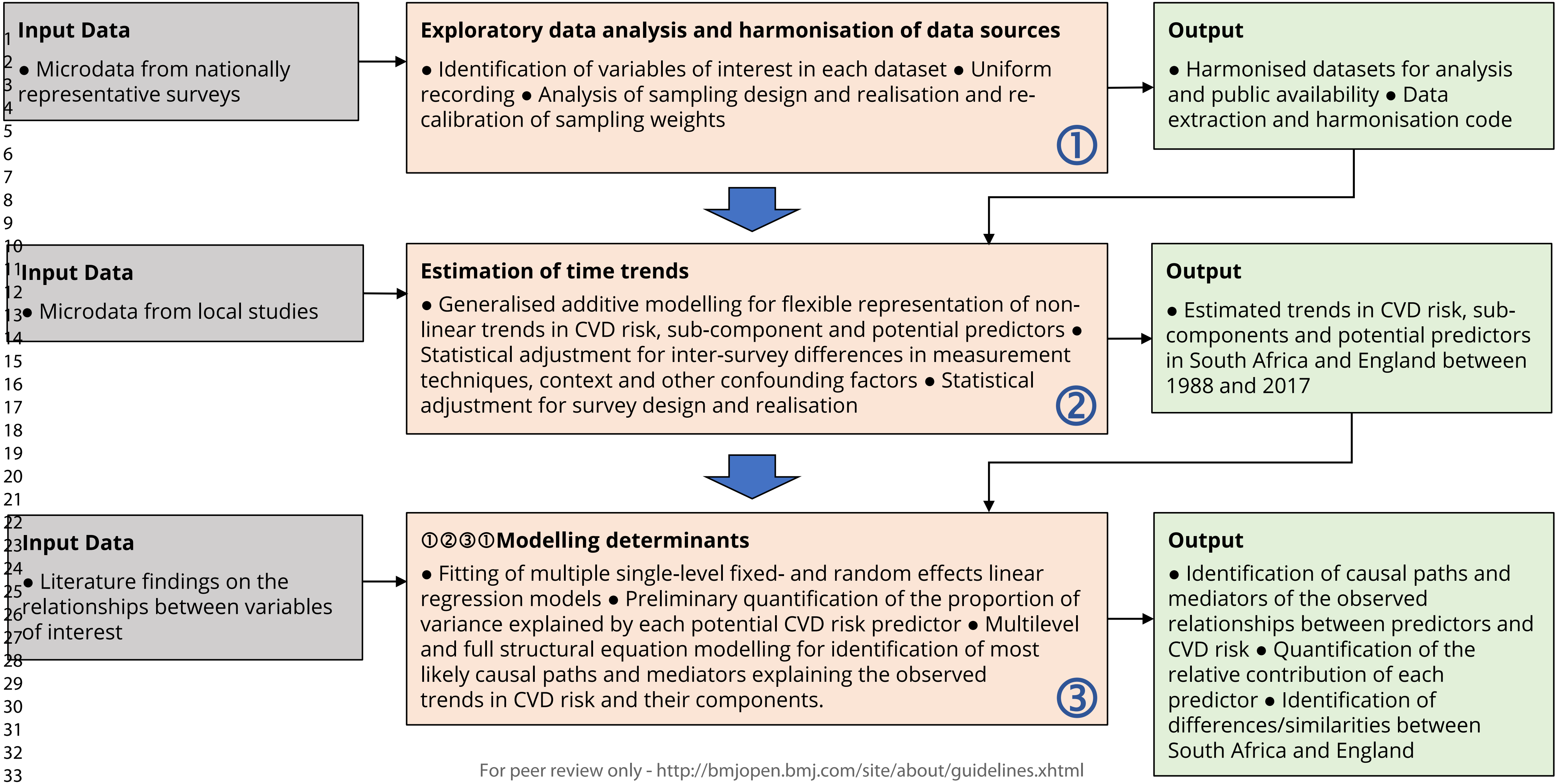
Input Data
• Microdata from nationally representative surveys
Exploratory data analysis and harmonisation of data sources
• Identification of variables of interest in each dataset • Uniform recording • Analysis of sampling design and realisation and re-calibration of sampling weights
①
Output
• Harmonised datasets for analysis and public availability • Data extraction and harmonisation code
Input Data
• Microdata from local studies
Estimation of time trends
• Generalised additive modelling for flexible representation of non-linear trends in CVD risk, sub-component and potential predictors • Statistical adjustment for inter-survey differences in measurement techniques, context and other confounding factors • Statistical adjustment for survey design and realisation
②
Output
• Estimated trends in CVD risk, sub-components and potential predictors in South Africa and England between 1988 and 2017
Input Data
• Literature findings on the relationships between variables of interest
①②③①Modelling determinants
• Fitting of multiple single-level fixed- and random effects linear regression models • Preliminary quantification of the proportion of variance explained by each potential CVD risk predictor • Multilevel and full structural equation modelling for identification of most likely causal paths and mediators explaining the observed trends in CVD risk and their components.
③
Output
• Identification of causal paths and mediators of the observed relationships between predictors and CVD risk • Quantification of the relative contribution of each predictor • Identification of differences/similarities between South Africa and England

The RECORD statement – checklist of items, extended from the STROBE statement, that should be reported in observational studies using routinely collected health data.

	Item No.	STROBE items	Location in manuscript where items are reported	RECORD items	Location in manuscript where items are reported
**Title and abstract**					
	1	(a) Indicate the study's design with a commonly used term in the title or the abstract (b) Provide in the abstract an informative and balanced summary of what was done and what was found	Abstract	RECORD 1.1: The type of data used should be specified in the title or abstract. When possible, the name of the databases used should be included.<br><br>RECORD 1.2: If applicable, the geographic region and timeframe within which the study took place should be reported in the title or abstract.<br><br>RECORD 1.3: If linkage between databases was conducted for the study, this should be clearly stated in the title or abstract.	Abstract<br><br>Abstract<br><br>N/A
**Introduction**					
Background rationale	2	Explain the scientific background and rationale for the investigation being reported	Introduction – Background; Abstract		
Objectives	3	State specific objectives, including any prespecified hypotheses	Introduction – Study aims		
**Methods**					
Study Design	4	Present key elements of study design early in the paper	Methods and Analysis		
Setting	5	Describe the setting, locations, and relevant dates, including periods of recruitment, exposure, follow-up, and data collection	Methods and Analysis – Sample and data sources		

For peer review only

Participants	6	*(a) Cohort study* - Give the eligibility criteria, and the sources and methods of selection of participants. Describe methods of follow-up<br>*Case-control study* - Give the eligibility criteria, and the sources and methods of case ascertainment and control selection. Give the rationale for the choice of cases and controls<br>*Cross-sectional study* - Give the eligibility criteria, and the sources and methods of selection of participants<br><br>*(b) Cohort study* - For matched studies, give matching criteria and number of exposed and unexposed<br>*Case-control study* - For matched studies, give matching criteria and the number of controls per case	Methods and Analysis – Sample and data sources	RECORD 6.1: The methods of study population selection (such as codes or algorithms used to identify subjects) should be listed in detail. If this is not possible, an explanation should be provided.<br><br>RECORD 6.2: Any validation studies of the codes or algorithms used to select the population should be referenced. If validation was conducted for this study and not published elsewhere, detailed methods and results should be provided.<br><br>RECORD 6.3: If the study involved linkage of databases, consider use of a flow diagram or other graphical display to demonstrate the data linkage process, including the number of individuals with linked data at each stage.	Methods and Analysis – Sample and data sources
Variables	7	Clearly define all outcomes, exposures, predictors, potential confounders, and effect modifiers. Give diagnostic criteria, if applicable.	Methods and Analysis – Measurement	RECORD 7.1: A complete list of codes and algorithms used to classify exposures, outcomes, confounders, and effect modifiers should be provided. If these cannot be reported, an explanation should be provided.	
Data sources/ measurement	8	For each variable of interest, give sources of data and details of methods of assessment (measurement).<br>Describe comparability of assessment methods if there is more than one group	Methods and Analysis – Analysis plan		

For peer review only

Bias	9	Describe any efforts to address potential sources of bias			
Study size	10	Explain how the study size was arrived at	Methods and Analysis – Sample and data sources & Table 1		
Quantitative variables	11	Explain how quantitative variables were handled in the analyses. If applicable, describe which groupings were chosen, and why	N/A (Protocol. Analysis to be done.)		
Statistical methods	12	(a) Describe all statistical methods, including those used to control for confounding<br>(b) Describe any methods used to examine subgroups and interactions<br>(c) Explain how missing data were addressed<br>(d) *Cohort study* - If applicable, explain how loss to follow-up was addressed<br>*Case-control study* - If applicable, explain how matching of cases and controls was addressed<br>*Cross-sectional study* - If applicable, describe analytical methods taking account of sampling strategy<br>(e) Describe any sensitivity analyses	Methods and Analysis – Analysis plan		
Data access and cleaning methods		..		RECORD 12.1: Authors should describe the extent to which the investigators had access to the database population used to create the study population.	Methods and Analysis – Sample and data sources

For peer review only

				RECORD 12.2: Authors should provide information on the data cleaning methods used in the study.	Methods and Analysis – Analysis plan
Linkage		..		RECORD 12.3: State whether the study included person-level, institutional-level, or other data linkage across two or more databases. The methods of linkage and methods of linkage quality evaluation should be provided.	Methods and Analysis – Sample and data sources
**Results**					
Participants	13	(a) Report the numbers of individuals at each stage of the study (*e.g.*, numbers potentially eligible, examined for eligibility, confirmed eligible, included in the study, completing follow-up, and analysed)<br>(b) Give reasons for non-participation at each stage.<br>(c) Consider use of a flow diagram	N/A (Protocol. Analysis to be done.)	RECORD 13.1: Describe in detail the selection of the persons included in the study (*i.e.,* study population selection) including filtering based on data quality, data availability and linkage. The selection of included persons can be described in the text and/or by means of the study flow diagram.	N/A (Protocol. Analysis to be done.)
Descriptive data	14	(a) Give characteristics of study participants (*e.g.*, demographic, clinical, social) and information on exposures and potential confounders<br>(b) Indicate the number of participants with missing data for each variable of interest<br>(c) *Cohort study* - summarise follow-up time (*e.g.*, average and total amount)	N/A (Protocol. Analysis to be done.)		
Outcome data	15	*Cohort study* - Report numbers of outcome events or summary measures over time<br>*Case-control study* - Report numbers in each exposure	N/A(Protocol. Analysis to be done.)		

For peer review only

		category, or summary measures of exposure<br>*Cross-sectional study* - Report numbers of outcome events or summary measures			
Main results	16	(a) Give unadjusted estimates and, if applicable, confounder-adjusted estimates and their precision (e.g., 95% confidence interval). Make clear which confounders were adjusted for and why they were included<br>(b) Report category boundaries when continuous variables were categorized<br>(c) If relevant, consider translating estimates of relative risk into absolute risk for a meaningful time period	N/A (Protocol. Analysis to be done.)		
Other analyses	17	Report other analyses done—e.g., analyses of subgroups and interactions, and sensitivity analyses	Methods and Analysis – Analysis plan		
**Discussion**					
Key results	18	Summarise key results with reference to study objectives	N/A (Protocol. Analysis to be done.)		
Limitations	19	Discuss limitations of the study, taking into account sources of potential bias or imprecision. Discuss both direction and magnitude of any potential bias	N/A (Protocol. Analysis to be done.)	RECORD 19.1: Discuss the implications of using data that were not created or collected to answer the specific research question(s). Include discussion of misclassification bias, unmeasured confounding, missing data, and changing eligibility over time, as they pertain to the study being reported.	N/A (Protocol. Analysis to be done.)

For peer review only

Interpretation	20	Give a cautious overall interpretation of results considering objectives, limitations, multiplicity of analyses, results from similar studies, and other relevant evidence	N/A (Protocol. Analysis to be done.)		
Generalisability	21	Discuss the generalisability (external validity) of the study results	N/A (Protocol. Analysis to be done.)		
**Other Information**					
Funding	22	Give the source of funding and the role of the funders for the present study and, if applicable, for the original study on which the present article is based	Funding		
Accessibility of protocol, raw data, and programming code		..		RECORD 22.1: Authors should provide information on how to access any supplemental information such as the study protocol, raw data, or programming code.	N/A (Protocol. Analysis to be done.)

*Reference: Benchimol EI, Smeeth L, Guttmann A, Harron K, Moher D, Petersen I, Sørensen HT, von Elm E, Langan SM, the RECORD Working Committee. The REporting of studies Conducted using Observational Routinely-collected health Data (RECORD) Statement. *PLoS Medicine* 2015; in press.

*Checklist is protected under Creative Commons Attribution (CC BY) license.

For peer review only

Explaining population trends in cardiovascular risk: protocol for a comparative analysis of health transitions in South Africa and England using nationally representative survey data

Journal:	*BMJ Open*
Manuscript ID	bmjopen-2022-061034.R1
Article Type:	Protocol
Date Submitted by the Author:	14-Feb-2022
Complete List of Authors:	Adjaye-Gbewonyo, Kafui; University of Greenwich, Faculty of Education, Health and Human Sciences<br>Cois, Annibale; Stellenbosch University Faculty of Medicine and Health Sciences, Division of Health Systems and Public Health
**Primary Subject Heading**:	Public health
Secondary Subject Heading:	Cardiovascular medicine, Global health, Research methods
Keywords:	Cardiac Epidemiology < CARDIOLOGY, EPIDEMIOLOGY, PUBLIC HEALTH, SOCIAL MEDICINE

SCHOLARONE™
Manuscripts

Title

Explaining population trends in cardiovascular risk: protocol for a comparative analysis of health transitions in South Africa and England using nationally representative survey data

Kafui Adjaye-Gbewonyo[1], Annibale Cois[2]

[1] Institute for Lifecourse Development, Faculty of Education, Health and Human Sciences, School of Human Sciences, University of Greenwich, London, UK.

[2] Division of Health Systems and Public Health, Department of Global Health, Stellenbosch University, Tygerberg, South Africa

Corresponding Author: Dr Kafui Adjaye-Gbewonyo, K.AdjayeGbewonyo@greenwich.ac.uk, Faculty of Education, Health and Human Sciences, School of Human Sciences, Dreadnought Building, Old Royal Naval College, Park Row, London, SE10 9LS

Word count: 3607

For peer review only

Abstract

Introduction Cardiovascular diseases (CVD) are the leading causes of death globally and share determinants with other major non-communicable diseases. Risk factors for CVD are routinely measured in population surveys and thus provide an opportunity to study health transitions. Understanding the drivers of health transitions in countries that have not followed expected paths compared to those that exemplified models of 'epidemiologic transition', such as England, can generate knowledge on where resources may best be directed to reduce the burden of disease. This study aims to examine notions of epidemiologic transition by identifying and quantifying the drivers of change in CVD risk in a middle-income African setting compared to a high-income European setting.

Methods and analysis This is a secondary joint analysis of data collected within the scope of multiple population surveys conducted in South Africa and England between 1998 and 2017 on nationally representative samples of the adult population. The study will use a validated, non-laboratory risk score to estimate and compare the distribution of and trends in total CVD risk in the population. Statistical modelling techniques (fixed-effects and random-effects multilevel regression models and structural equation models) will be used to examine how various factors explain the variation in CVD risk over time in the two countries.

Ethics and dissemination This study has obtained approval from the University of Greenwich (20.5.6.8) and Stellenbosch University (X21/09/027) Research Ethics Committees. It uses anonymised microdata originating from population surveys which received ethical approval by the relevant bodies, with no additional primary data collection. Results of the study will be disseminated through (1) peer-reviewed articles in open access journals; (2) policy briefs; (3) conferences and meetings; and (4) public engagement activities designed to reach health professionals, governmental bodies, civil society and the lay public. A harmonised dataset will be made publicly available through online repositories.

Strengths and limitations of this study

- The study assesses a composite score of CVD risk in addition to individual risk factors.
- Trends will be examined over a nearly 20-year period and the contributions of a range of factors to these trends will be quantified at multiple levels.
- Comparative analysis will explore health transitions in different contexts.
- Innovative structural equation modelling techniques help to account for variation across surveys.
- Source data are representative but heterogeneous.

For peer review only

INTRODUCTION

Background

In his influential paper, 'Sick Individuals, Sick Populations',[1] British epidemiologist Geoffrey Rose posed two questions: 'Why do some individuals have hypertension?' and 'Why do some populations have much hypertension, whilst in others it is rare?' He wrote that while we might gain complete understanding of the first question—why individuals vary in their disease risk—we may miss 'the most important public health question',[1] namely why populations have different disease burdens. As Rose noted, oftentimes what makes some individuals sicker than others is not necessarily the same as what makes some populations 'sicker' than others.[1] Understanding how a population's health is changing over time is one way to address this important second question and better understand the epidemiology of disease.

In our current era, non-communicable diseases (NCDs) have become the leading causes of death and illness globally. These conditions are often referred to as 'chronic diseases', having long duration, requiring continuing care, and often viewed as not infectious or directly transmissible to others.[2] In 1971, Abdul Omran proposed a theory of epidemiologic transition in which societies shift from having a disease profile marked by undernutrition and infection ('age of pestilence and famine') to a period where these conditions decrease ('age of receding pandemics') and finally toward a stage dominated by chronic NCDs as life expectancies and economies improve ('age of degenerative and man-made diseases').[3,4]

The theory of epidemiologic transition contends that this rise in NCDs such as cardiovascular disease (CVD) is caused by a number of factors. These include demographic changes, specifically the ageing of the population as mortality declines and life expectancy rises, given that the risk for many chronic diseases increases with age. In addition, decreased morbidity and mortality from infections, undernutrition and maternal and neonatal causes have also been implicated in the demographic changes leading to the rise of chronic NCDs, as medical care, sanitation and standards of living improve. 'Lifestyle' and behavioural changes such as reduced physical activity and worsening diets linked to urbanisation and rising economic status, have also been hypothesised to contribute to the growing NCD 'epidemic'. Thus, epidemiologic transition is seen to parallel and be a product of several other transitions, including demographic, fertility, technologic and nutritional transitions. These transitions are assumed to be occurring globally, having first occurred in industrialised, high-income countries (HICs) such as the UK while Sub-Saharan African countries have typically been viewed as being at an earlier stage along this path.[3,4]

However, there have been critiques as to whether this theory is in fact a valid model of epidemiologic change.[5] Although Omran's model has been updated over the years, many exceptions to its predictions have been noted.[4-8] For example, recent focus has emphasized the so-called 'double burden of disease' in low- and middle-income countries (LMICs), representing a time in which infectious diseases are still highly prevalent, yet chronic NCDs are simultaneously rising.[6] This is currently the case in South Africa where the HIV/AIDS and tuberculosis (TB) epidemic, combined with other communicable and nutritional diseases, NCDs and violence/injury has even been characterised as a 'quadruple disease burden'.[9]

Not only has the epidemiologic transition theory been criticised for being an inaccurate model of transition in many LMICs, including in the African region,[10] scholars such as Simon Szreter and Alexander Mercer have also contended that it may not be an accurate description of the health transitions that occurred in the HICs on which the theory was built, including England.[11-13] These

For peer review only

and other studies suggest that 'epidemiologic transition' may not be the simple answer to explain changing disease patterns, underscoring the need for more detailed analyses examining other factors as well as additional research at the country level.[7]

However, empirical research to study the underlying demographic, socio-economic and other determinants of epidemiological changes is lacking,[3] and detailed case studies of health transition are particularly needed from the African continent.[6]

This study aims to address this need through a comparative study of South Africa and England, using CVD risk as a case study of health transitions. South Africa is a middle-income African country and former British colony whose history of colonialism and apartheid has led to enduring structural and racial inequalities. England is a high-income country whose position as a former imperial power has contributed to its comparative wealth and industrialisation. This history also ties England to South Africa. For our analysis, we draw on socio-ecological and ecosocial frameworks to identify potential explanatory variables of interest at the individual (biological, behavioural, socio-economic, demographic) and contextual (environmental, societal) levels.[14,15]

This project expands on previous work by Cois examining hypertension trends in South Africa.[16-18] In his work, he found that data from the National Income Dynamics Study suggested that hypertension levels have been decreasing since the late 2000s,[16] more in line with the trends in HICs than with those in many LMICs.[19,20] This was in spite of increasing levels of overweight/obesity and other risk factors for hypertension and was not fully explained by antihypertensive medication use.

Building on this work, this study will extend the analysis beyond blood pressure (BP) and hypertension, and examine and compare trends of overall cardiovascular risk in South Africa. The study will also attempt to identify which factors (demographic, economic, health/medical, environmental, psychosocial, behavioural) explain the changes in risk and to what extent. Thus, we will empirically test the relative contributions of factors implicated in epidemiologic transition, including ageing, improved living standards, urbanisation, lifestyle change. In addition, we expand on the types of explanatory factors that have been previously examined in Cois's work to fill in gaps and examine unanswered questions about potential drivers of change in population health. Therefore, we will incorporate not only demographic, behavioural, socioeconomic and healthcare variables that are traditionally considered as risk factors for CVD, but crucially we will also focus on other social determinants of health that have been overlooked in the research, including psychosocial, environmental and contextual factors. We will also compare these trends in cardiovascular risk and its drivers in South Africa to those in England, where mortality from CVD has been noted to be declining in recent decades due to increased prevention and treatment.[21]

Study Aims

The aim of this study is to utilise existing empirical data to understand the demographic, behavioural, social and environmental drivers of recent health transitions in a middle-income African country with a high infectious disease burden compared to a HIC with a low infectious disease burden, using CVD risk as a case study. Understanding changes in population risk for chronic disease will lead to improved public health policy and prevention strategies.

The study also aims to produce a harmonised dataset compiling national surveys measuring CVD risk factors in South Africa for others to use in future research as well as code for harmonising and merging the England surveys.

The research questions are as follows:

For peer review only - http://bmjopen.bmj.com/site/about/guidelines.xhtml

1. What are the population trends in CVD risk in South Africa since its first national health survey in 1998?
2. To what extent are these trends explained by demographic, behavioural, social, environmental, health-related and/or other factors?
3. How do these results compare to those in a high-income country with a different infectious disease profile such as England over the same time period?

We hypothesise that overall CVD risk increased in South Africa and decreased in England during the study period but that these changes are not fully explained by demographic, socioeconomic, behavioural or treatment changes that often form part of epidemiological transition theory. We hypothesise that other social and environmental factors as well as infectious disease interactions may have contributed to some of the patterns in CVD risk.

METHODS AND ANALYSIS

This is a secondary joint analysis of data collected within the scope of populations surveys conducted in South Africa and England between 1998 and 2017 on nationally representative samples of the adult population.

Sample and data sources

We will draw our main sample from repeated cross-sections of nationally-representative surveys in South Africa and England that include information on chronic-disease related conditions and risk factors, including BP readings and anthropometric measurements. Population-based surveys serve as a valuable data source to address questions regarding epidemiologic change, particularly in LMICs where data from health facilities and registries are often incomplete and not representative of the wider population. National health and social surveys provide a unique opportunity to examine these questions, as they often include indicators of chronic disease risk that can easily be measured in the field such as BP and height, weight and waist circumference which can be used to measure obesity.

Data for South Africa will be drawn from the Demographic and Health Surveys (DHS), National Income Dynamics Study (NIDS), South Africa National Health and Nutrition Examination Survey (SANHANES) and Study on Global Ageing and Adult Health (SAGE).[22-26] Taken together, these datasets cover 11 cross-sections of the South African adult population spanning a 19-year period from 1998 to 2017. A description of the samples in these publicly-available datasets is presented in Table 1. Local data such as from South African Health and Demographic Surveillance Sites (HDSSs) will be used to validate and inform the main analysis.[27] In addition, we will derive area-level and environmental variables from South Africa's censuses (1996, 2001 and 2011),[28,29] inter-census Community Surveys (2007 and 2016),[29-31] District Health Barometer [32] and IPUMS Terra,[33] among other sources.

Data for England will be drawn from the Health Survey for England (HSE) over the same time period of 1998 to 2017 (Table 1).[34,35] Area-level variables derived from the UK Census, IPUMS Terra and other sources will be constructed to analyse contextual effects.

For peer review only

Table 1. National Surveys for Analysis

South African Surveys				England Surveys			
Survey	Year	Adult Ages	Sample Size	Survey	Year	Adult Ages	Sample Size
DHS	1998	15+	13 827	HSE	1998	16+	15 908
				HSE	1999	16+	14 642
				HSE	2000	16+	10 481
				HSE	2001	16+	15 647
				HSE	2002	16+	10 330
DHS	2003	15+	8 115	HSE	2003	16+	14 836
				HSE	2004	16+	13 520
				HSE	2005	16+	10 303
				HSE	2006	16+	14 142
SAGE	2007-8	18+	4 223	HSE	2007	16+	6 882
NIDS	2008	15+	16 872	HSE	2008	16+	15 098
				HSE	2009	16+	4 645
NIDS	2010-11	15+	21 874	HSE	2010	16+	8 420
NIDS	2012	15+	22 457	HSE	2011	16+	8 610
SANHANES	2012	15+	7 436 [a]	HSE	2012	16+	8 290
NIDS	2014-15	15+	22 741	HSE	2013	16+	8 795
SAGE	2014-15	18+ [b]	26 804	HSE	2014	16+	8 077
				HSE	2015	16+	8 034
DHS	2016	15+	5 685	HSE	2016	16+	8 011
NIDS	2017	15+	30 109	HSE	2017	16+	7 997

[a] Sample completing the physical exam; [b] Population-representative sample aged 50+ years, and a control sample aged 18-49 years.

HSE = Health Survey for England; DHS = Demographic and Health Survey; SAGE = Study on Global Ageing and Adult Health; NIDS = National Income Dynamics Study; SANHANES = South Africa National Health and Nutrition Examination Survey.

All national datasets being used are publicly available from the data owners upon registration/ application, apart from the South African DHS 2003 which has been obtained from the National Department of Health, Special Licence data and removed variables for the HSE which are being requested from the UK Data Service and NatCen Social Research, as well as select local datasets which will be requested from the relevant HDSSs.

To be consistent across surveys, we will restrict our samples to adults aged 18 years and above. However, sensitivity analyses using other age groups such as ages 16 and over or ages 50+ will also be conducted for comparison.

Measurements

Outcome

The main outcome of interest (cardiovascular risk) will be quantified using Gaziano's non-laboratory based CVD risk score.[36] CVD risk scores such as the Framingham risk scores [37] are widely used in clinical and other settings to predict risk of having a future CVD event based on a series of laboratory results (such as lipid profiles) and other demographic and self-reported data. Non-laboratory-based measures were developed for use in low-resource settings where laboratory measures may be costly or impossible to obtain. Among those, Gaziano's score expresses the risk of developing any

For peer review only

cardiovascular disease event in the following 5-years as a function of average systolic blood pressure (SBP), body mass index (BMI), age, sex, smoking status, current treatment for hypertension and diabetes diagnosis.[36] It has been tested in South African samples and classified over 90% of men and over 94% of women similarly to the Framingham risk score.[38] The use of alternative risk predictive models will also be investigated prior to the final analyses. In addition, we will examine individual CVD risk components such as BP, BMI, smoking, hypertension treatment and diabetes diagnosis as secondary outcomes.

Explanatory variables

To assess trends in CVD risk over time, survey year will be the main time variable. To explain and decompose any time trends observed, demographic (age), socioeconomic (household assets, education, occupation, household size), health behaviour/'lifestyle' (physical activity, smoking, alcohol consumption, dietary factors) and geographic (urban/rural), as well as variables relating to other health states or conditions (TB, reproductive history for women), medications and healthcare utilisation will be considered.

Given South Africa's unique history of racial segregation and noted racial/ethnic disparities in England, racial ascription (with its historical categorisation into four socially defined 'population groups' in South Africa and 'ethnicities' in England) will be also be included among the model predictors/effect modifiers as an indicator of racial discrimination. We will consider psychosocial factors by incorporating social support where available or proxies (such as marital status) and stress indicators (such as resting heart rate and household death) that are available across surveys.[39-43]

In addition, drawing on ecosocial theory and social determinants of health frameworks,[14,26] we will incorporate environmental and contextual variables from IPUMS Terra[33] and other local sources, such as climate data (monthly temperatures) and land use data. These will be analysed at the regional/provincial and district council levels and at smaller levels (municipality, enumeration area) where possible. We will also construct other area-level variables, such as measures of inequality, area deprivation and indicators of health services and status from censuses, community surveys, the District Health Barometer and other government sources.

Analysis plan

Stata v. 17 (StataCorp, College Station, TX) and R Statistical software v. 4.0 (R Core Team, Vienna, Austria) will be used for data management, preliminary analyses and reporting. Mplus v. 8 (Muthén & Muthén, Los Angeles, CA) and MLwiN v. 2.02 (Centre for Multilevel Modelling, Bristol, UK) will be used for path analysis, multilevel and structural modelling. Statistical analyses will be performed separately by gender, given that gender differences in CVD risk are notable[18, 37] and that reproductive factors such as pregnancy status and hormonal contraceptive use are also associated with BP and BMI. In the final stage of the study, gender-specific results will be combined to estimate to what extent changes in gender distribution across age categories and time are contributing to average population CVD risk trends.

The pooled analysis of the available datasets will include three phases (Figure 1). Our approach draws from methods used in demography and epidemiology (public health) as well as econometric and psychometric methods:

1. In the first phase, exploratory data analysis will identify the relevant variables and ensure, with appropriate recoding and/or preliminary elaboration if necessary, the congruence of the values across datasets. The sampling designs of each survey will be analysed and compared, in order to recover the information needed to produce a congruent set of pooled sampling weights and

For peer review only

identify adequate methods to take into account the effect of clustering and stratification on the precision of the estimates. Uniform re-calibration of sampling weights to match a consistent set of age-gender-specific population totals will be also considered, if justified by the observed inter-survey discrepancies.

2. In the second phase, we will recover time trends for the outcome variable (CVD risk), its sub-components (age, SBP, smoking status, BMI, hypertension treatment, diabetes diagnosis) and the hypothesised predictors (socioeconomic, behavioural, psychosocial, infectious-disease-related, contextual, etc.). Additive models including spline representation of the relationship of the outcome with time will be used to take into account the possibility of non-linear trends. Relevant covariates will be included to adjust for inter-survey differences in measurement and possible confounding factors (e.g. seasonal differences in measurement on blood pressure)
3. In the third phase, fixed-effects, random-effects and more general structural equation modelling (SEM) methods will be used to examine the relationships between the outcomes of interest and their hypothesised predictors, in order to identify potential determinants of the observed trends in CVD risk and its components. These methods have been chosen because they allow us to quantify and estimate the contributions of various factors to the overall variation in CVD risk over time.

   Single-level fixed- and random-effects linear regression models will be first used to examine the association between CVD risk, time and other potential predictors at individual level, and to quantify the proportion of variance explained by the different variables. Multilevel and full structural equation models will be then used to analyse more in detail these relationships at individual and area-levels and to identify most likely causal paths and mediators explaining the observed trends in CVD risk and their components. The use of full structural models, including latent variables to represent quantities measured with error builds and extends Dr Cois's methodological innovations of using SEM and latent variable approaches to analyse BP while accounting for potential measurement error and variation across surveys.[17] The approach has not been used often in health research, but is a promising method to test causal hypotheses.

In all analyses, the complex sampling design of each survey will be taken into account with the methods developed during the first phase of the study, so that 1) the estimates produced by the models will refer to the total populations of South Africa and England at each time point, and 2) the estimates of standard errors and other measures of uncertainty are adjusted for the actual realisation of the sampling design. Missingness will be addressed through the use of both complete-case analyses as well as through methods treating missingness on the outcomes as missing at random, conditional on covariates. We will run a series of models using different sets of variables. Sensitivity analyses accounting for data quality through quality scores may also be conducted.[18]

Data and code management

A secure cloud storage system will be set up to store the original datasets as acquired from the data providers. Access to the storage area will be restricted to the study investigators, to fulfil limitations regarding data sharing as required by the data provider. Relevant metadata (including survey protocols, questionnaires and data collection procedures) will also be stored jointly with the data.

All recoding and analysis activities will be documented through Stata (.do) and/or R script files, to ensure reproducibility. A GitHub (GitHub Inc, San Francisco, CA) private repository will be set up to ensure automatic versioning of all code produced for the study and facilitate collaboration between investigators.

For peer review only

Patient and public involvement

A stakeholder and user advisory group has been created for this study, consisting of members from research and academia, government, healthcare and civil society organisations. The group is providing input and guidance on the study analysis and dissemination of the findings and members of the group will be involved in the co-production of various study outputs.

ETHICS AND DISSEMINATION

Ethics

This study has obtained Ethics exemption from the University Research Ethics Committee at the University of Greenwich (Ref. 20.5.6.8, 20/06/2021) and approval from the Health Research Ethics Committee at Stellenbosch University (Ref. X21/09/027, 28/09/2021).

This project is a secondary data analysis involving the use of anonymised/de-identified microdata from population samples, and no additional primary data will be collected. All projects from which the data being used in this analysis originate received prior ethical approval by the relevant bodies and explicit consent/assent was obtained from all participants.

The study will follow the terms and conditions of the data providers, including using the data only for the purposes set out in the project, not attempting to identify individuals in the data and preserving confidentiality, citing the datasets and acknowledging and informing the data providers in and about all outputs resulting from the data. Data about the individuals in the datasets will be derived solely from the existing anonymised/de-identified datasets, and no additional information about individuals will be added to the data. Therefore, we do not foresee any added risk of identification. We will follow the framework for research ethics established by the UK Economic and Social Research Council (ESRC) which funds the study, including seeking to maximise the benefits and minimise the harms of the research, respecting the rights and dignity of research subjects, upholding the integrity and transparency of the research, defining lines of responsibility and accountability and maintaining the independence of the research. We will also ensure that results of our analysis are reported accurately and responsibly.

Dissemination

The findings of the study will be disseminated through multiple avenues, namely (1) peer-reviewed articles in open access scientific journals; (2) policy briefs for health policymakers at the national level as well as a report targeting the public sector, civil society and health professions; (3) presentations at conferences and meetings; (4) public engagement activities and a report launch in order to reach health professionals, governmental bodies, civil society and the lay public.

Within the limitations of the data sharing agreements, a harmonised dataset of the pooled national surveys for South Africa which will be produced and made publicly available through major online data repositories, namely DataFirst at the University of Cape Town (https://www.datafirst.uct.ac.za/), the Human Sciences Research Council in Pretoria (http://datacuration.hsrc.ac.za/) and the UK Data service (https://ukdataservice.ac.uk/).

The dataset will include all the variables of interest used for the study, uniformly recoded to facilitate use for future studies in South Africa. To facilitate the re-use and sharing of the data, a narrative document describing all the variable definitions and derived variables created from each dataset will be drafted by the investigators to accompany the datasets for deposit. This will include citations for

For peer review only

all source data used in the creation of the datasets. The code used to harmonise and merge the Health Surveys for England and South African surveys will also be made available through public repositories. A variable list and codebook will be created for both the England and South Africa final datasets. A series of workshops targeting researchers, students, policymakers and other subjects will be organised to provide training on the optimal use of the harmonised datasets.

REFERENCES

1. Rose G. Sick individuals and sick populations. *Int J Epidemiol* 2001;30(3):427-32.
2. Adjaye-Gbewonyo K, Vaughan M. Reframing NCDs? An analysis of current debates. *Glob Health Action* 2019;12(1), DOI: 10.1080/16549716.2019.1641043.
3. Santosa A, Wall S, Fottrell E, et al. The development and experience of epidemiological transition theory over four decades: a systematic review. *Glob Health Action* 2014;7:23574.
4. Omran AR. The Epidemiologic Transition: A Theory of the Epidemiology of Population Change. *Milbank Mem Fund Q* 1971;49(4):509-38.
5. Vaughan, M, Adjaye-Gbewonyo, K, Mika, M (eds.). Epidemiological Change and Chronic Disease in Sub-Saharan Africa: Social and historical perspectives. London: UCL Press; 2021 [Available from: https://doi.org/10.14324/111.9781787357044]
6. Agyei-Mensah S, de-Graft Aikins A. Epidemiological transition and the double burden of disease in Accra, Ghana. *J Urban Health* 2010;87(5):879-97.
7. Murray CJL, Barber RM, Foreman KJ, et al. Global, regional, and national disability-adjusted life years (DALYs) for 306 diseases and injuries and healthy life expectancy (HALE) for 188 countries, 1990–2013: quantifying the epidemiological transition. *The Lancet* 2015;386(10009):2145-91.
8. Kabudula CW, Houle B, Collinson MA, et al. Progression of the epidemiological transition in a rural South African setting: findings from population surveillance in Agincourt, 1993-2013. *BMC Public Health* 2017;17(1):424.
9. Pillay-van Wyk V, Msemburi W, Laubscher R, et al. Mortality trends and differentials in South Africa from 1997 to 2012: second National Burden of Disease Study. *The Lancet Global Health* 2016;4(9):e642-e53.
10. Vaughan M. Conceptualising metabolic disorder in Southern Africa: Biology, history and global health. *BioSocieties* 2018;14(1):123–42.
11. Szreter S, Mooney G. Urbanization, Mortality, and the Standard of Living Debate: New Estimates of the Expectation of Life at Birth in Nineteenth-century British Cities. *The Economic History Review* 1998;51(1):84-112.
12. Szreter S. Industrialization and health. *Br Med Bull* 2004;69:75-86.
13. Mercer A. Infections, chronic disease, and the epidemiological transition: A new perspective. Rochester: University of Rochester Press; 2014.
14. Krieger N. Proximal, distal, and the politics of causation: What's level got to do with it? *American Journal of Public Health* 2008;98(2):221-30.

For peer review only

15. Krieger N. Epidemiology and the people's health: Theory and context. New York: Oxford University Press; 2011.

16. Cois A, Ehrlich R. Antihypertensive treatment and blood pressure trends among South African adults: A repeated cross-sectional analysis of a population panel survey. *PLoS One* 2018;13(8):e0200606.

17. Cois A, Ehrlich R. Analysing the socioeconomic determinants of hypertension in South Africa: a structural equation modelling approach. *BMC Public Health* 2014;14:414.

18. Cois A. Understanding blood pressure dynamics in the South African population: a latent variables approach to the analysis and comparison of data from multiple surveys (Doctoral dissertation]. Cape Town: University of Cape Town; 2017. [Available from: https://open.uct.ac.za/handle/11427/25196]

19. Danaei G, Finucane MM, Lin JK, et al. National, regional, and global trends in systolic blood pressure since 1980: systematic analysis of health examination surveys and epidemiological studies with 786 country-years and 5·4 million participants. *The Lancet* 2011;377(9765):568-77.

20. Zhou B, Bentham J, Di Cesare M, et al. Worldwide trends in blood pressure from 1975 to 2015: a pooled analysis of 1479 population-based measurement studies with 19.1 million participants. *The Lancet* 2017;389(10064):37-55.

21. Guzman-Castillo M, Ahmed R, Hawkins N, et al. The contribution of primary prevention medication and dietary change in coronary mortality reduction in England between 2000 and 2007: a modelling study. *BMJ Open* 2015;5:e006070.

22. Department of Health Republic of South Africa. South Africa Demographic and Health Survey 1998: Full Report. Pretoria: Department of Health; 2002.

23. National Income Dynamics Study. Website. [Available from: http://www.nids.uct.ac.za. Accessed: October 2021].

24. Shisana O, Labadarios D, Rehle T, et al. South African National Health and Nutrition Examination Survey (SANHANES-1). Cape Town: HSRC Press; 2013.

25. World Health Organization. Health statistics and information systems: WHO Study on global AGEing and adult health (SAGE). [Available from: http://www.who.int/healthinfo/sage/en/. Accessed: October 2021]

26. Kowal P, Chatterji S, Naidoo N, et al. Data resource profile: the World Health Organization Study on global AGEing and adult health (SAGE). *Int J Epidemiol* 2012;41(6):1639-49.

27. SAPRIN. South African Population Research Infrastructure Network [Available from: http://saprin.mrc.ac.za/about.html. Accessed: October 2021]

28. Statistics South Africa. Census 2011 Statistical Release - P0301.4. Pretoria: Stats SA; 2012.

29. Minnesota Population Center. Integrated Public Use Microdata Series, International: Version 7.2 [Machine-Readable Database]. Minneapolis: University of Minnesota; 2019.

30. Statistics South Africa. Community Survey, 2007 (revised version). Pretoria: Stats SA; 2007.

31. Statistics South Africa. Community Survey 2016, Statistical release P0301. Pretoria: Stats SA; 2016.

For peer review only

32. Massyn N, Tanna G, Day C, et al. District Health Barometer: District Health Profiles 2017/18. Durban: Health Systems Trust; 2018.

33. Ruggles S, Manson SM, Kugler TA, et al. IPUMS Terra: Integrated Data on Population and Environment. 2 ed. Minneapolis, MN: IPUMS; 2018.

34. Health Survey for England London: UCL; 2019 [Available from: https://www.ucl.ac.uk/epidemiology-health-care/research/epidemiology-and-public-health/research/health-and-social-surveys-research-group/studies-0. Accessed: October 2021]

35. Mindell J, Biddulph JP, Hirani V, et al. Cohort profile: the health survey for England. *Int J Epidemiol* 2012;41(6):1585-93.

36. Gaziano TA, Young CR, Fitzmaurice G, et al. Laboratory-based versus non-laboratory-based method for assessment of cardiovascular disease risk: the NHANES I Follow-up Study cohort. *The Lancet* 2008;371(9616):923-31.

37. Damen JA, Pajouheshnia R, Heus P, et al. Performance of the Framingham risk models and pooled cohort equations for predicting 10-year risk of cardiovascular disease: a systematic review and meta-analysis. *BMC Med* 2019;17(109).

38. Gaziano TA, Pandya A, Steyn K, et al. Comparative assessment of absolute cardiovascular disease risk characterization from non-laboratory-based risk assessment in South African populations. *BMC Med* 2013;11:170.

39. Adjaye-Gbewonyo K, Avendano M, Subramanian SV, et al. Income inequality and depressive symptoms in South Africa: A longitudinal analysis of the National Income Dynamics Study. *Health & Place* 2016;42:37-46.

40. Adjaye-Gbewonyo K, Kawachi I, Subramanian SV, et al. High social trust associated with increased depressive symptoms in a longitudinal South African sample. *Social Science & Medicine* 2018;197:127-35.

41. Adjaye-Gbewonyo K, Kawachi I, Subramanian SV, et al. Income inequality and cardiovascular disease risk factors in a highly unequal country: a fixed-effects analysis from South Africa. *Int J Equity Health* 2018;17(1):31.

42. Cois A, Day C. Obesity trends and risk factors in the South African adult population. *BMC Obesity* 2015;2:42.

43. Cois A, Ehrlich R. Socioeconomic Status Modifies the Seasonal Effect on Blood Pressure: Findings from a National Panel Study. *Medicine* 2015;94(35):e1389.

Acknowledgments

The authors thank Megan Vaughan [University College London (UCL)], Ed Fottrell (UCL), Luis Rego (UCL), Debbie Bradshaw [South African Medical Research Council (SAMRC)] and Jané Joubert (SAMRC) for their valuable comments on drafts of this protocol.

For peer review only

Authors' contributions

The study was conceived by KA, who prepared the first draft of the protocol. AC contributed in refining the scope of the study, the analysis, dissemination and data sharing plans. Both authors read and approved the final protocol. KA and AC will conduct variable selection and harmonisation across datasets; implement the analysis strategy and run the analyses; draft and revise reports/publications resulting from the analyses; implement research impact and stakeholder involvement activities. KA will ensure overall coordination of the research activities as the PI of the study, in collaboration with AC as the co-PI.

Funding

This work is supported by UKRI Economic and Social Research Council grant number ES/V003259/1.

Competing interests

None declared.

For peer review only

FIGURE CAPTIONS

Figure 1: Data analysis plan

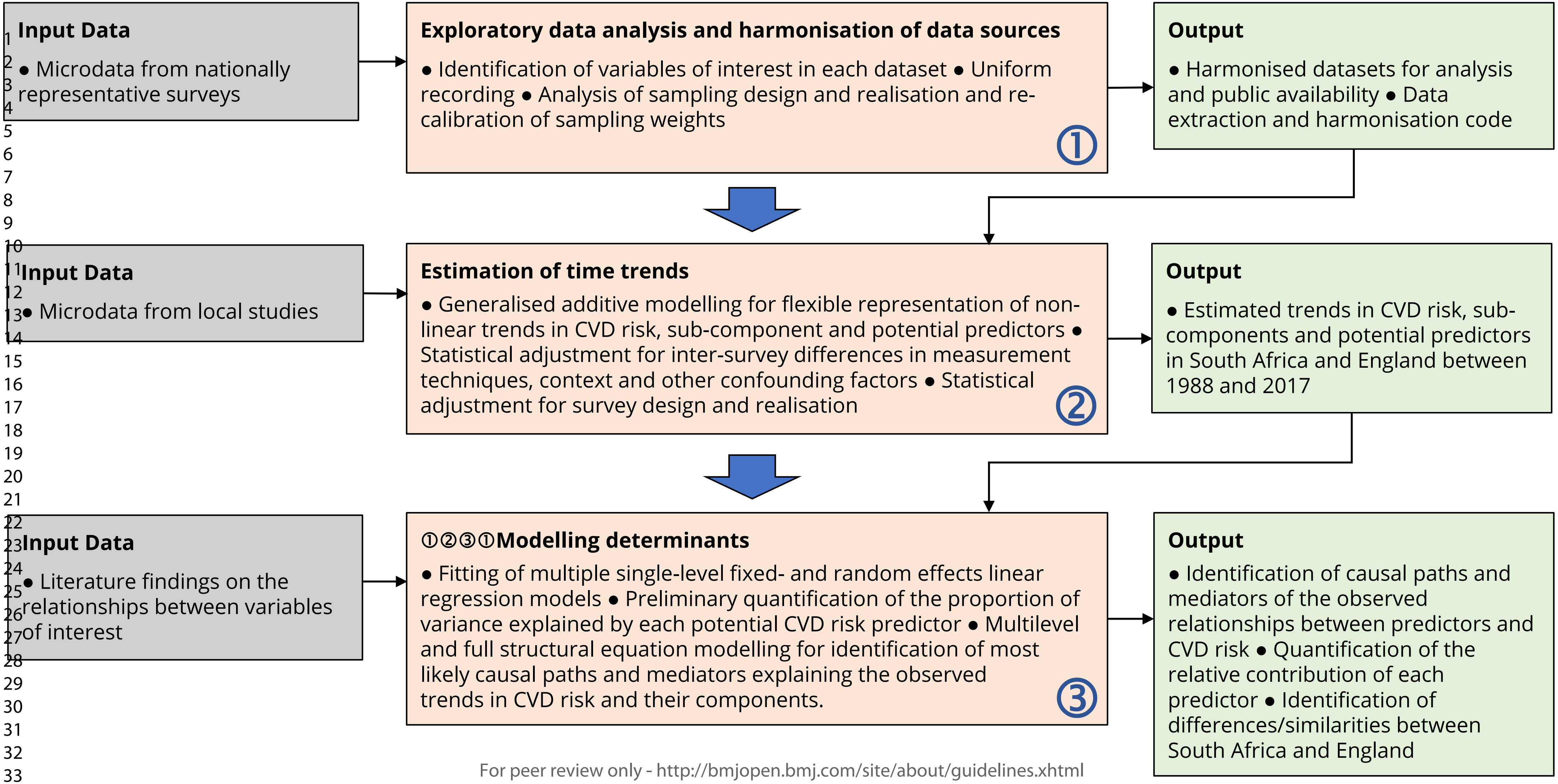
Input Data
● Microdata from nationally representative surveys
Exploratory data analysis and harmonisation of data sources
● Identification of variables of interest in each dataset ● Uniform recording ● Analysis of sampling design and realisation and re-calibration of sampling weights
①
Output
● Harmonised datasets for analysis and public availability ● Data extraction and harmonisation code
Input Data
● Microdata from local studies
Estimation of time trends
● Generalised additive modelling for flexible representation of non-linear trends in CVD risk, sub-component and potential predictors ● Statistical adjustment for inter-survey differences in measurement techniques, context and other confounding factors ● Statistical adjustment for survey design and realisation
②
Output
● Estimated trends in CVD risk, sub-components and potential predictors in South Africa and England between 1988 and 2017
Input Data
● Literature findings on the relationships between variables of interest
①②③①Modelling determinants
● Fitting of multiple single-level fixed- and random effects linear regression models ● Preliminary quantification of the proportion of variance explained by each potential CVD risk predictor ● Multilevel and full structural equation modelling for identification of most likely causal paths and mediators explaining the observed trends in CVD risk and their components.
③
Output
● Identification of causal paths and mediators of the observed relationships between predictors and CVD risk ● Quantification of the relative contribution of each predictor ● Identification of differences/similarities between South Africa and England

The RECORD statement – checklist of items, extended from the STROBE statement, that should be reported in observational studies using routinely collected health data.

	Item No.	STROBE items	Location in manuscript where items are reported	RECORD items	Location in manuscript where items are reported
**Title and abstract**					
	1	(a) Indicate the study's design with a commonly used term in the title or the abstract (b) Provide in the abstract an informative and balanced summary of what was done and what was found	Abstract	RECORD 1.1: The type of data used should be specified in the title or abstract. When possible, the name of the databases used should be included.<br><br>RECORD 1.2: If applicable, the geographic region and timeframe within which the study took place should be reported in the title or abstract.<br><br>RECORD 1.3: If linkage between databases was conducted for the study, this should be clearly stated in the title or abstract.	Abstract<br><br>Abstract<br><br>N/A
**Introduction**					
Background rationale	2	Explain the scientific background and rationale for the investigation being reported	Introduction – Background; Abstract		
Objectives	3	State specific objectives, including any prespecified hypotheses	Introduction – Study aims		
**Methods**					
Study Design	4	Present key elements of study design early in the paper	Methods and Analysis		
Setting	5	Describe the setting, locations, and relevant dates, including periods of recruitment, exposure, follow-up, and data collection	Methods and Analysis – Sample and data sources		

For peer review only

Participants	6	*(a) Cohort study* - Give the eligibility criteria, and the sources and methods of selection of participants. Describe methods of follow-up<br>*Case-control study* - Give the eligibility criteria, and the sources and methods of case ascertainment and control selection. Give the rationale for the choice of cases and controls<br>*Cross-sectional study* - Give the eligibility criteria, and the sources and methods of selection of participants<br><br>*(b) Cohort study* - For matched studies, give matching criteria and number of exposed and unexposed<br>*Case-control study* - For matched studies, give matching criteria and the number of controls per case	Methods and Analysis – Sample and data sources	RECORD 6.1: The methods of study population selection (such as codes or algorithms used to identify subjects) should be listed in detail. If this is not possible, an explanation should be provided.<br><br>RECORD 6.2: Any validation studies of the codes or algorithms used to select the population should be referenced. If validation was conducted for this study and not published elsewhere, detailed methods and results should be provided.<br><br>RECORD 6.3: If the study involved linkage of databases, consider use of a flow diagram or other graphical display to demonstrate the data linkage process, including the number of individuals with linked data at each stage.	Methods and Analysis – Sample and data sources
Variables	7	Clearly define all outcomes, exposures, predictors, potential confounders, and effect modifiers. Give diagnostic criteria, if applicable.	Methods and Analysis – Measurement	RECORD 7.1: A complete list of codes and algorithms used to classify exposures, outcomes, confounders, and effect modifiers should be provided. If these cannot be reported, an explanation should be provided.	
Data sources/ measurement	8	For each variable of interest, give sources of data and details of methods of assessment (measurement).<br>Describe comparability of assessment methods if there is more than one group	Methods and Analysis – Analysis plan		

For peer review only

Bias	9	Describe any efforts to address potential sources of bias			
Study size	10	Explain how the study size was arrived at	Methods and Analysis – Sample and data sources & Table 1		
Quantitative variables	11	Explain how quantitative variables were handled in the analyses. If applicable, describe which groupings were chosen, and why	N/A (Protocol. Analysis to be done.)		
Statistical methods	12	(a) Describe all statistical methods, including those used to control for confounding<br>(b) Describe any methods used to examine subgroups and interactions<br>(c) Explain how missing data were addressed<br>(d) *Cohort study* - If applicable, explain how loss to follow-up was addressed<br>*Case-control study* - If applicable, explain how matching of cases and controls was addressed<br>*Cross-sectional study* - If applicable, describe analytical methods taking account of sampling strategy<br>(e) Describe any sensitivity analyses	Methods and Analysis – Analysis plan		
Data access and cleaning methods		..		RECORD 12.1: Authors should describe the extent to which the investigators had access to the database population used to create the study population.	Methods and Analysis – Sample and data sources

For peer review only

				RECORD 12.2: Authors should provide information on the data cleaning methods used in the study.	Methods and Analysis – Analysis plan
Linkage		..		RECORD 12.3: State whether the study included person-level, institutional-level, or other data linkage across two or more databases. The methods of linkage and methods of linkage quality evaluation should be provided.	Methods and Analysis – Sample and data sources
**Results**					
Participants	13	(a) Report the numbers of individuals at each stage of the study (*e.g.*, numbers potentially eligible, examined for eligibility, confirmed eligible, included in the study, completing follow-up, and analysed)<br>(b) Give reasons for non-participation at each stage.<br>(c) Consider use of a flow diagram	N/A (Protocol. Analysis to be done.)	RECORD 13.1: Describe in detail the selection of the persons included in the study (*i.e.,* study population selection) including filtering based on data quality, data availability and linkage. The selection of included persons can be described in the text and/or by means of the study flow diagram.	N/A (Protocol. Analysis to be done.)
Descriptive data	14	(a) Give characteristics of study participants (*e.g.*, demographic, clinical, social) and information on exposures and potential confounders<br>(b) Indicate the number of participants with missing data for each variable of interest<br>(c) *Cohort study* - summarise follow-up time (*e.g.*, average and total amount)	N/A (Protocol. Analysis to be done.)		
Outcome data	15	*Cohort study* - Report numbers of outcome events or summary measures over time<br>*Case-control study* - Report numbers in each exposure	N/A(Protocol. Analysis to be done.)		

For peer review only

		category, or summary measures of exposure<br>*Cross-sectional study* - Report numbers of outcome events or summary measures			
Main results	16	(a) Give unadjusted estimates and, if applicable, confounder-adjusted estimates and their precision (e.g., 95% confidence interval). Make clear which confounders were adjusted for and why they were included<br>(b) Report category boundaries when continuous variables were categorized<br>(c) If relevant, consider translating estimates of relative risk into absolute risk for a meaningful time period	N/A (Protocol. Analysis to be done.)		
Other analyses	17	Report other analyses done—e.g., analyses of subgroups and interactions, and sensitivity analyses	Methods and Analysis – Analysis plan		
**Discussion**					
Key results	18	Summarise key results with reference to study objectives	N/A (Protocol. Analysis to be done.)		
Limitations	19	Discuss limitations of the study, taking into account sources of potential bias or imprecision. Discuss both direction and magnitude of any potential bias	N/A (Protocol. Analysis to be done.)	RECORD 19.1: Discuss the implications of using data that were not created or collected to answer the specific research question(s). Include discussion of misclassification bias, unmeasured confounding, missing data, and changing eligibility over time, as they pertain to the study being reported.	N/A (Protocol. Analysis to be done.)

For peer review only

Interpretation	20	Give a cautious overall interpretation of results considering objectives, limitations, multiplicity of analyses, results from similar studies, and other relevant evidence	N/A (Protocol. Analysis to be done.)		
Generalisability	21	Discuss the generalisability (external validity) of the study results	N/A (Protocol. Analysis to be done.)		
**Other Information**					
Funding	22	Give the source of funding and the role of the funders for the present study and, if applicable, for the original study on which the present article is based	Funding		
Accessibility of protocol, raw data, and programming code		..		RECORD 22.1: Authors should provide information on how to access any supplemental information such as the study protocol, raw data, or programming code.	N/A (Protocol. Analysis to be done.)

*Reference: Benchimol EI, Smeeth L, Guttmann A, Harron K, Moher D, Petersen I, Sørensen HT, von Elm E, Langan SM, the RECORD Working Committee. The REporting of studies Conducted using Observational Routinely-collected health Data (RECORD) Statement. *PLoS Medicine* 2015; in press.

*Checklist is protected under Creative Commons Attribution (CC BY) license.

For peer review only
