# Supplementary material for: Explaining population trends in cardiovascular risk: protocol for a comparative analysis of health transitions in South Africa and England using nationally representative survey data
Source: BMJ Open. 2022 Mar 29;12(3):e061034. doi: 10.1136/bmjopen-2022-061034 (PMC8966565; doi:10.1136/bmjopen-2022-061034)
Supplement: Reviewer comments [file bmjopen-2022-061034.reviewer_comments.pdf]

PEER REVIEW HISTORY

BMJ Open publishes all reviews undertaken for accepted manuscripts. Reviewers are asked to complete a checklist review form (**http://bmjopen.bmj.com/site/about/resources/checklist.pdf**) and are provided with free text boxes to elaborate on their assessment. These free text comments are reproduced below.

ARTICLE DETAILS

**TITLE (PROVISIONAL)**	Explaining population trends in cardiovascular risk: protocol for a comparative analysis of health transitions in South Africa and England using nationally representative survey data
**AUTHORS**	Adjaye-Gbewonyo, Kafui; Cois, Annibale

This article was not externally reviewed at BMJ Open. Protocols that have been independently assessed prior to submission to BMJ Open are usually fast-tracked to publication on the grounds that further substantial changes will not be possible. This independent assessment will usually be external, independent review for both a substantial grant award from a non-commercial or government funder and ethics approval.
